# Copper (I)-Chloroquine Complexes: Interactions with DNA and Ferriprotoporphyrin, Inhibition of β-Hematin Formation and Relation to Antimalarial Activity

**DOI:** 10.3390/ph15080921

**Published:** 2022-07-25

**Authors:** Wilmer Villarreal, William Castro, Sorenlis González, Marylin Madamet, Rémy Amalvict, Bruno Pradines, Maribel Navarro

**Affiliations:** 1 Grupo de Química Inorgânica Medicinal e Reações Aplicadas, Instituto de Química, Universidade Federal do Rio Grande do Sul (UFRGS), Porto Alegre 91501-970, Brazil; wilmer.villarreal@ufrgs.br; 2 Centro de Química, Instituto Venezolano de Investigaciones Científicas (IVIC), Caracas 1020-A, Venezuela; wcastro10@gmail.com (W.C.); sorenlis@hotmail.com (S.G.); 3 Unité Parasitologie et Entomologie, Département Microbiologie et Maladies Infectieuses, Institut de Recherche Biomédicale des Armées, 13005 Marseille, France; mmadamet@gmail.com (M.M.); remy_alt@yahoo.fr (R.A.); bruno.pradines@gmail.com (B.P.); 4 Aix Marseille Univ, IRD, SSA, AP-HM, VITROME, 13005 Marseille, France; 5 IHU Méditerranée Infection, 13005 Marseille, France; 6 Centre National de Référence du Paludisme, 13005 Marseille, France; 7 Laboratório de Química Bioinorgânica e Catálise, Departamento de Química, Instituto de Ciências Exatas, Universidade Federal de Juiz de Fora (UFJF), Juiz de Fora 36036-900, Brazil

**Keywords:** copper (I) complexes, antimalarial metallodrugs, malarial molecular targets

## Abstract

A new Cu(I)-chloroquine (CQ) complex [Cu(CQ)(PPh_3_)_2_]NO_3_ (**1**) was synthesized and characterized, and its mechanism of action studied concomitant with the previously reported complex [Cu(CQ)_2_]Cl (**2**). These copper (I) coordination compounds can be considered as potential antimalarial agents because they show better inhibition of the CQ-resistant strain in in vitro studies than CQ alone. In comparison with other metal-CQ complexes, only the gold complex was similar to (**1**), i.e., more active than CQ against both CQ-susceptible (3D7) and CQ-resistant strains (W2). These two copper (I)-compounds also demonstrated higher antiplasmodial activity against W2 than other copper complexes reported to date. This suggests that the incorporation of the copper metal center enhanced the biological activity of CQ. To better understand their significant growth inhibition of the *Plasmodium falciparum* parasite, the interaction with two essential molecular targets for the survival and proliferation of the malarial parasite were studied. These were the ferriprotoporphyrin group and the DNA, both important targets for current antimalarial drugs at the asexual erythrocytic stages. Both compounds (**1**,**2**) exhibited significant interactions with these targets. In particular, interactions with the DNA were dominated by the intercalator properties of the CQ ligand but may have also been affected by the presence of copper. Overall, these compounds were better parasitic inhibitors than chloroquine diphosphate (CQDP) alone or other previously reported metal-CQ complexes such as platinum, ruthenium and gold.

## 1. Introduction

**Figure 1:**
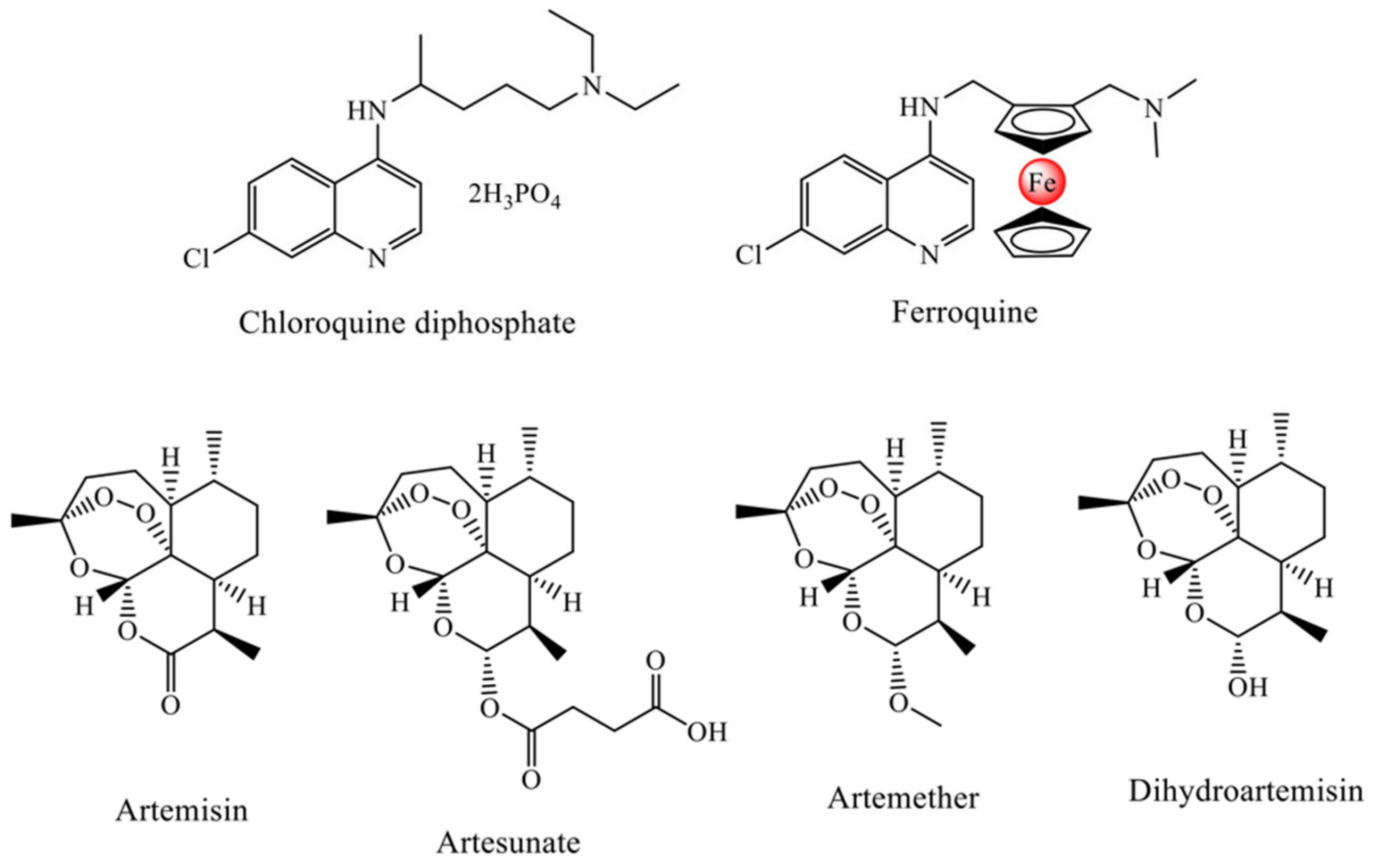
Drugs recommended by the WHO for malaria treatments and ferroquine (Phase II).

Malaria is a public health concern of tragic proportions, with an estimated 241 million new cases and 627,000 deaths globally in 2020, with most of the victims children under 5 years of age, [1]. The effectiveness of available antiplasmodial drugs against the blood stage of the infection is increasingly threatened by the emergence and spread of drug-resistant parasite strains. Chloroquine diphosphate (CQDP), the cornerstone antimalarial drug for many decades, has become largely ineffective against *P. falciparum*, the deadliest malaria parasite, due to the drug-resistance of that strain [2]. The current frontline malaria treatments recommended by the WHO are artemisinin-based combination therapies (ACTs) with a treatment success rate above 95%. These are the combinations of artemisinin, or its derivatives (artesunate, artemether, dihydroartemisinin), and longer acting antiplasmodial drugs with distinct modes of action to prevent the development of drug-resistance (Figure 1). However, the parasite is now also becoming more resistant to these treatments [3,4]. Therefore, researchers are exploring the therapeutic potential of metal-based antimalarial agents, especially since the discovery of ferroquine (Figure 1), once the most potent organometallic antimalarial drug [5].

Several in vivo studies have indicated increased activity of biologically active compounds when bound to specific metals [6,7,8]. Our team previously demonstrated that the complexation of a metal ion (e.g., Rh [9], Ru [10], and Au [11]) with CQ led to significant modification of the physicochemical properties of the free CQ, and this resulted in enhanced activity against CQ-resistant strains of *Plasmodium*. It was also assumed that complexes with endogenous metals (Co, Cu, Zn, and Fe) could be less toxic than those with non-essentials metals [12]. Copper-containing coordination compounds were found to be promising therapeutic agents in a diverse range of diseases, including malaria, because of their ability to act by various mechanisms, such as inhibition of proteasome activity [13], telomerase activity [14], reactive oxygen species (ROS) formation [15], DNA degradation [16], and DNA intercalation [17], among others. One example of the success of copper complexes against various diseases is the phase I clinical trial with casiopeinas for cancer treatment [18].

Developments regarding the use of novel copper complexes as antimalarial agents have been reported. Padhye showed that conjugation of carboxamidrazone ligands by copper enhanced their antiplasmodial activities against *P. falciparum* 3D7 strain [19]. Habluetzel reported two water-soluble Cu(I) phosphonate complexes [20] and their activity against *Plasmodium* in the sporogonic stage. Recently, Parra’s group reported the synthesis and considerable antimalarial activity of two copper compounds with [(7-chloroquinolin-4-yl)amino]acetophenones. They demonstrated that complexation with Cu(II) increased the activity of free ligands against *P. falciparum* [21].

Based on the above, we evaluated the anti-plasmodial activity of two chloroquine-copper (I) complexes in vitro, and studied the possible mechanism of action using two known and essential molecular targets for the survival of the malarial parasite such as hemozoin formation and DNA [22,23,24,25].

## 2. Results and Discussion

### 2.1. Synthesis of Copper Complexes

**Scheme 1:**
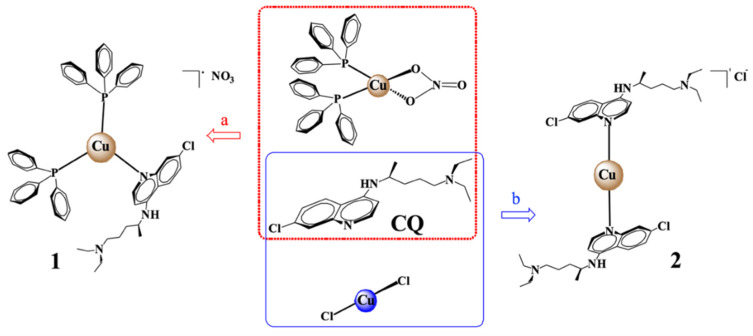
Methodology for the synthesis of copper(I)-chloroquine complexes **1** and **2**. (**a**) Acetonitrile/reflux 1 h-[Cu(PPh_3_)_2_NO_3_]:CQ 1:1.2. (**b**) Acetonitrile/reflux 1 h-CuCl_2_:CQ 1:2.2.

The synthesis of complex **1** involved the reaction of the precursor [Cu(PPh_3_)_2_NO_3_] dissolved in acetonitrile with an excess of CQ under an inert atmosphere and refluxed for one hour. Cu(CQ)(PPh_3_)_2_]NO_3_ (**1**) was isolated as pale-yellow solid in good yield (72%). The characterization techniques indicated the presence of the phosphine and the CQ in the copper complex (Scheme 1). Analysis of the nuclear magnetic resonance (NMR) (Figures S1–S6) obtained in one-dimensional (1D) and two-dimensional (2D) correlation spectroscopy (COSY), heteronuclear multiple quantum coherence (HMQC) and heteronuclear multiple bond coherence (HMBC) experiments, showed relation between the phosphine and CQ ligand in 2:1 ratio. Furthermore, the changes in the chemical shifts in the ^1^H and ^13^C{^1^H} NMR signals between the spectra of the complex **1** and the free ligand (CQ) gave indication of the CQ coordination mode (Table S1). The structure of the quinoline region underwent great modifications after the ligand coordinated to the metal center, suggesting CQ coordination via the quinolinic nitrogen. 

**Figure 2:**
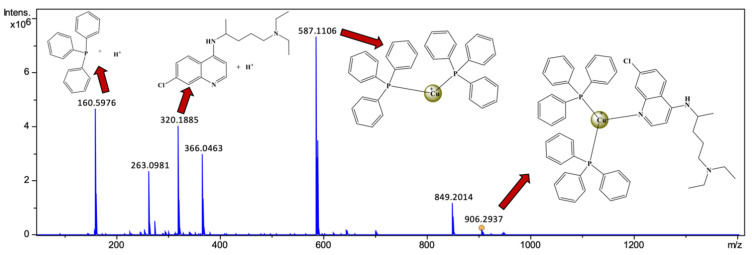
ESI(+)-MS spectrum of [Cu(CQ)(PPh_3_)_2_]NO_3_ (**1**) in acetonitrile.

The ESI(+)-MS spectrum (Figure 2) exhibited the following signals: the molecular ion [M−NO_3_]^+^, its fragmentations with the loss of the chloroquine ligand [M−CQ−NO_3_]^+^, and those for the free ligands [CQ + H]^+^ and [PPh_3_ + H]^+^. Theoretical and experimental isotopic distributions for **1** were in very good agreement (Figure S7a,b). The molar conductivity value was obtained in the interval for electrolyte 1:1 and indicated the ${\mathrm{NO}}_{3}^{-}$ group as counter ion. The electron paramagnetic resonance (EPR) spectrum of the complex did not result in any signal, which was an indication of the absence of paramagnetic species, and confirmed the oxidation state of copper (I) (Figure S8). Complex **1** is proposed as tricoordinate Cu(I) complex with trigonal planar geometry, where one chloroquine and two PPh_3_ groups are coordinated to Cu(I) (Scheme 1). This geometry is often stabilized by bulky ligands, typically phosphines, phenanthrolines or quinolones [26]. The synthesis of complex **2** was as previously reported by us [27].

The stability of compound **1** and **2** was evaluated in a mixture of 90% DMSO-*d_6_* and 10% PBS-*D_2_O* by ^1^H and ^31^P{^1^H} NMR spectroscopy at different times (0, 24 and 48 h). In parallel, their stability in a mixture of 10% DMSO and 90% PBS medium (pH 7.4) was also studied with UV-Visible spectrophotometry. No significant changes were observed for complex **2** (Figures S9 and S12b) in either study. In contrast, complex **1** displayed slight changes in both studies (Figures S10, S11 and S12a) that can either be attributed to π-π interactions between the chloroquine and phosphine ligands or due to possible oxidation of Cu(I) to Cu(II) under these conditions.

It is known that coordinated metals can enhance the efficacy of the organic drugs. This strategy has been used in the treatment of malaria with great success by many research groups, e.g., Singh [28], Biot [29], Brocard [30], among others. In specific, the teams of Sanchez-Delgado and Navarro proposed the modification of CQ through the incorporation of a transition metal into its molecular structure [9,10,11]. The purpose with these complexes **1** and **2** (Scheme 1) was to increase the biological activity of the chloroquine and fight against the CQ resistance of the parasites. There may be a synergistic effect between CQ with known antimalarial effects and copper that may reduce toxicity and widen its biological activity by potentially attacking other molecular targets.

The evaluation of the interaction of the complexes **1** and **2** with DNA seemed appropriate because of the natural interaction that copper exhibits within our body, and the possible reactions these metal complexes could have with this biomolecule [16,17]. This interaction could cause alterations of the DNA of the parasite which could lead to its death.

The interaction with ferriprotoporphyrin was studied because of the general acceptance that quinolones, such as CQ, target the catabolism of the host’s haemoglobin by the parasite. This takes place in the acidic ‘food vacuole’ [31] and specifically inhibits haemozoin formation [32]. Given that in drug resistant parasites CQ does not affect the haemozoin formation, it presents an excellent target for newly developed drug candidates.

To validate the findings the results were compared with the in vitro biological activity against 3D7 and W2 strains (CQ-susceptible and CQ-resistant strains, respectively).

### 2.2. DNA as a Target

For a long time, the DNA of the malarial parasite has been a great drug target [33]. This is because causing DNA damage can be a route to kill the parasite. DNA damage is also believed to be the mode of action of antimalarial drugs such as artemisinin [34] and chloroquine [24]. Metal-based drugs are well known to interact with DNA; therefore, a copper-CQ complex could have higher possibility of DNA interaction and an increased antimalarial activity. Experiments were designed and performed to study such interactions between the complexes **1** and **2** and DNA.

The interaction between the compounds and the DNA was studied by UV-Vis and fluorometric methods. The spectra of the copper(I)-CQ complexes in the presence of the DNA indicated reversive interaction. This was supported by the presence of two isosbestic points at 350 and 359 nm, indicating the emergence of new species.

**Figure 3:**
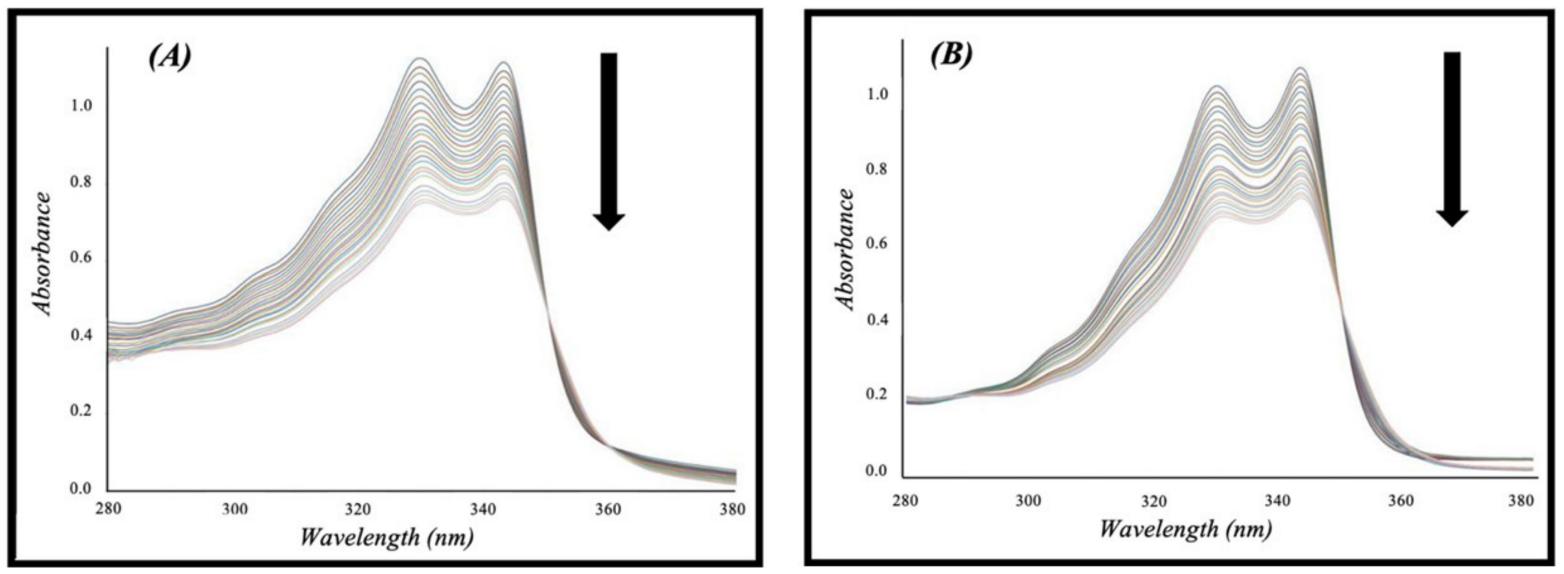
Spectrophotometric titration spectra of (**A**) [Cu(CQ)(PPh_3_)_2_]NO_3_ and (**B**) [Cu(CQ)_2_]Cl with CT-DNA.

The spectra for complexes **1** and **2** are shown in Figure 3 and the corresponding binding constants (Kb) calculated at 343 nm in Table 1. The Kb values lay within the range at which a compound is considered to be interacting with the DNA [35,36]. The results indicate stronger interaction between the DNA and the copper-chloroquine complexes than the CQDP alone under the same conditions. However, the Kb values of these compounds are not too different to other transition metal-CQ complexes [10].

**Figure 4:**
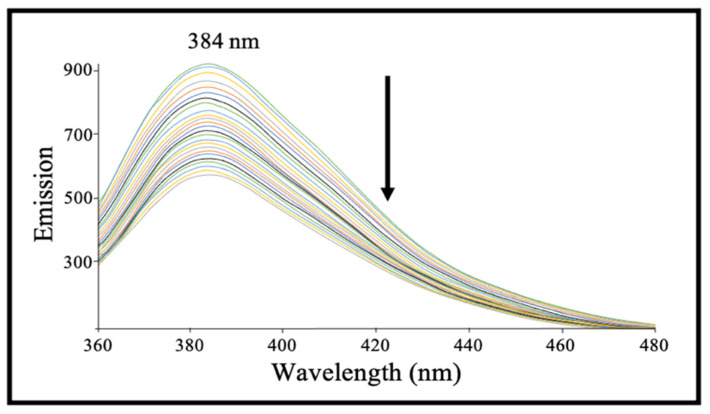
Fluorimetric titration spectra of [Cu(CQ)(PPh_3_)_2_]NO_3_ with CT-DNA. [Complex] = 2.24 × 10^−6^ M, [DNA] = 0–276 µM.

The intensity of the emission bands of complexes **1** and **2** reduces as the DNA concentration increases until saturation (see Figure 4 for complex **1**). This electronic perturbation in the emission spectra is caused by the interaction between the chloroquine-complexes and DNA. The binding constants (Kb_2_), shown in Table 1, were calculated using a Scatchard plot for the data at the emission maximum. The values are consistent with those calculated from the absorption studies and similar to other metal complexes [11,37]. However, they are an order of magnitude greater than the CQDP alone, indicating that these complexes interact with DNA stronger than the free CQDP.

**Figure 5:**
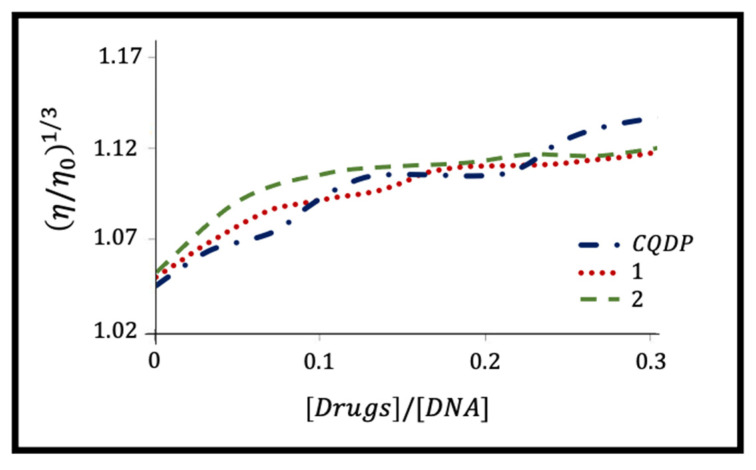
Effect of increasing concentration of [Cu(CQ)(PPh_3_)_2_]NO_3_, complex **1** (red dotted line), [Cu(CQ)_2_]Cl, complex **2** (green dash line) and CQDP (blue long dash line) on the relative viscosity of CT DNA at 25 °C.

The interaction between the Cu(I)-CQ complexes and DNA were further elucidated by hydrodynamics studies. The viscosity measurement is considered as a very efficient method to determine the binding mode of copper complexes to DNA (i.e., intercalation or non-intercalation), because of its sensitivity to the change in the length of the DNA double helix. The relative viscosity of CT-DNA increased in the presence of complex **1** and **2**, and also in the CQ free ligand (Figure 5). This suggests the lengthening of the double helix as the DNA base pairs intercalate the copper complexes. This finding is similar to that reported for other metal-CQ complexes and for ethidium bromide, which is a classical intercalator [38]. Thus, this supports the idea that complex **1** and **2** can act as DNA intercalator.

**Figure 6:**
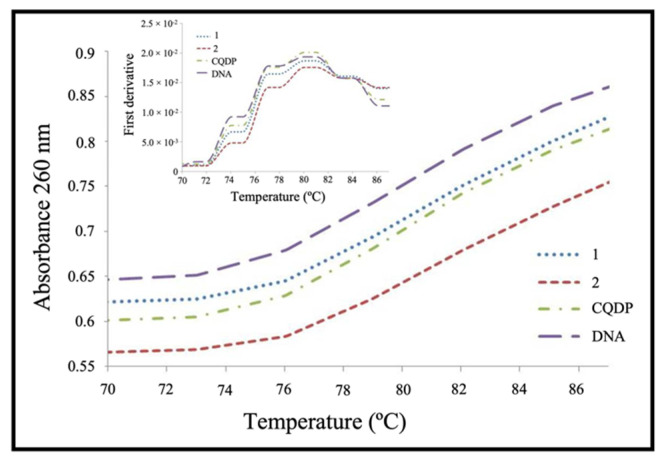
Thermal denaturation of CT-DNA alone (purple long dash line) and with the [Cu(CQ)(PPh_3_)_2_]NO_3_, complex **1** (blue dot line), [Cu(CQ)2]Cl, complex **2** (red dash line) and CQDP (green dash dot line) at a molar ratio, Ri = 0.05.

Molecules interacting with the DNA can affect its melting temperature (Tm), at which 50% of double stranded DNA changes to single stranded DNA. The thermal denaturation curves of DNA are shown in Figure 6. The free DNA melted at 80.7 °C. CQDP at a molar ratio (Ri) 0.05 (Table 1) had no effect; however, in higher ratios (Ri 0.1 and 0.2) it increased its melting temperature, ΔTm = +3.7 °C by stabilizing its double helix.

The complex **1** and **2** at Ri = 0.05 had a slight effect on the Tm of the DNA (ΔTm = −0.3 °C). A similar decrease in Tm values was also reported for other metal complexes, for example with *cis*-Pt(NH_3_)_2_Cl_2_ (ΔTm = −4.0 °C) [39] that is widely known to bond covalently with the DNA. When in a higher ratio (Ri 0.1 and 0.2), the profile curves of thermal denaturation were no longer comparable with those reported in the literature. Complex **1** and **2** may have severely destabilized the double helix.

A summary of the above studies suggests that the interactions between these copper (I)-CQ complexes with the DNA are dominated by the intercalator properties of the CQ ligand but may also be affected by the copper coordination to the CQ.

### 2.3. Interaction with Hemin and Inhibition of β-Hematin Formation

During blood stages, malaria parasites digest the host erythrocyte’s haemoglobin in order to complete its life cycle and survive. In this process heme is accumulated which is toxic to the parasite [40]. To avoid death, the parasite converts heme into the insoluble and inert hemozoin crystals in its digestive vacuole, becoming a target of action for some antimalarial agents; in fact, this is the most accepted mechanism of action of chloroquine [29].

**Figure 7:**
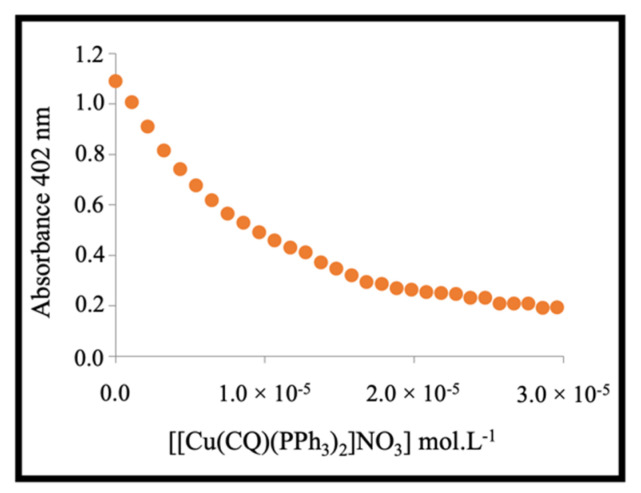
Fe(III)PPIX absorbance at 402 nm in the function of the concentration of complex **1**.

The Soret band was used to follow the interaction between the heme and complex **1** or **2** in the spectrophotometric titrations. This band refers to the absorption band of ferriprotoporphyrin IX (Fe(III)PPIX) with its maximum at 402 nm that is characteristic to the π-π * transitions in the porphyrin group. As an example, shown in Figure 7 for complex **1**, a hypochromic effect can be observed as consequence of the interaction between the heme and the compounds. The same behavior was also noticed by the complex **2** and the free CQ. All results for the complexes **1** and **2**, and for CQ are summarized in Table 2.

The calculated logK association constant (for 1:1 model) for CQDP (at pH 7.4, log K = 5.35 ± 0.03) was found to be similar to those reported elsewhere [41].

The values for complex **1** and **2** were calculated in an analogous manner at pH 7.4 (log K = 5.33 ± 0.10 and 3.55 ± 0.06, respectively). The similarity in the logK obtained for complex **1** and CQDP indicates similar interaction with hematin. Complex **2** has a weaker interaction then CQDP, but similar to mefloquine (LogK= 3.90 ± 0.08) [41].

**Figure 8:**
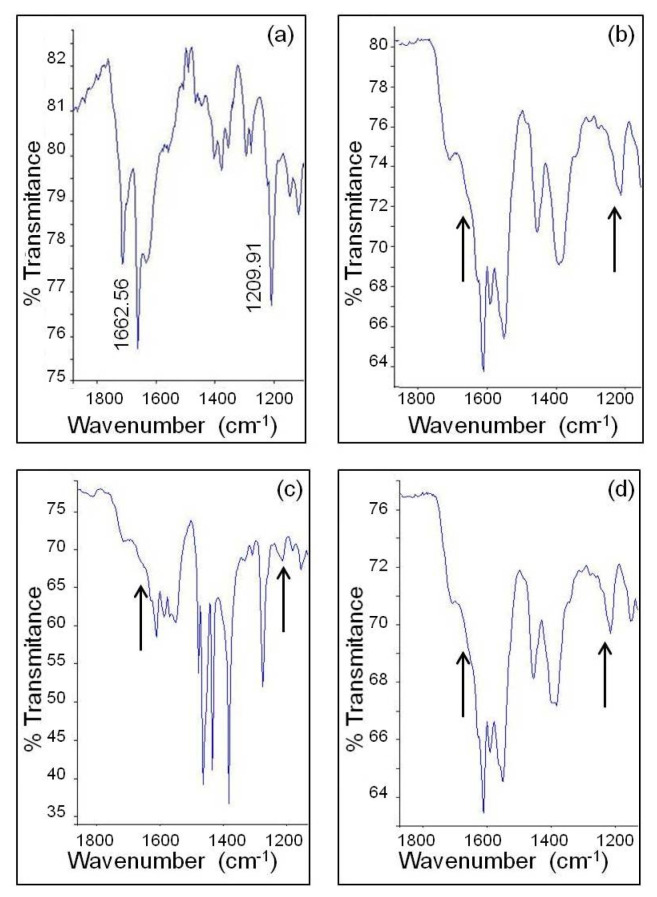
Infrared spectra of β-hematin obtained from hemin with characteristic β-hematin bands at 1660 and 1207 cm^−1^; (**a**) control experiment, in the presence of 3 equivalents of (**b**) CQDP, (**c**) [Cu(CQ)(PPh_3_)_2_]NO_3_ and (**d**) [Cu(CQ)_2_]Cl. In all cases the IR spectra were recorded after incubation at 60 °C, pH 5.0. The arrows in the spectra indicate the place of the characteristic β-hematin bands.

The inhibition of β-hematin formation by complex **1** and **2** was first evaluated qualitatively using FTIR spectroscopy by monitoring the changes in the characteristic β-hematin bands at 1660 and 1207 cm^−1^. Figure 8 shows the IR spectra recorded under the control conditions (a) and in the presence of three equivalents of CQDP (b), [Cu(CQ)(PPh_3_)_2_]NO_3_ (c) and [Cu(CQ)_2_]Cl (d). The disappearance of the IR bands at 1660 and 1210 cm^−1^ in Figure 8c,d suggests that both Cu-CQ complexes inhibited the β-hematin formation in a similar manner to CQDP (Figure 8b) and other known antiplasmodial drugs [42]. Next, their ability to inhibit heme aggregation was quantified using the method reported by Dominguez [43]; the results are presented in Table 2. Overall, these compounds exhibited better inhibition ability then CQDP alone. Furthermore, their inhibition ability of this important parasite target was also superior to other metal-CQ complexes such as platinum [44], ruthenium [45] and gold [11].

### 2.4. Antiplasmodial Activity

The antiplasmodial activity of the Cu(I)-CQ complexes was evaluated against CQ susceptible *Plasmodium falciparum* (3D7) and CQ resistant (W2) strains as shown in Table 3. Both copper complexes were more active than CQ alone against the CQ-resistant strain (W2), being up by 45 and 75% (relative activity index (RA) 1.75 and 1.45 for complex **1** and **2**, respectively. Against the CQ-susceptible strain (3D7), complex **1** showed similar results to CQ (RA 1.3). In comparison with other metal-CQ complexes reported previously [44,45] only the [AuCQPPh_3_]PF_6_ complex [11] showed similar activity to complex **1**, being more active than CQ in both CQ-susceptible and CQ-resistant strains. However, both complex **1** and **2** were up to 9 times more active against CQ resistant strains (W2) than the ruthenium analogue [Ru(η^6^-arene)(CQ)Cl_2_] [10], up to 3 times to Pt [44] and up to 2 times to Au [46]. This demonstrates the promising outlook for the copper-chloroquine complexes to eradicate this parasite. Moreover, these two compounds showed higher antiplasmodial activity against W2 (IC_50_ < 300 nM) than [(7-chloroquinolin-4-yl)amino]acetophenones and other copper complexes (IC_50_ ranged from 1 to 7 µM) [21].

The cell toxicity of these compounds was tested in J774 cells; the results are presented in Table 3. Free chloroquine and its copper(I) derivatives (**1** and **2**) presented less cytotoxicity than gentian violet used as a positive control. Based on the calculated selectivity index values (SI) complex **2** was the most selective (SI 106) against the CQ resistant strain (W2) but all compounds were promising against the *Plasmodium falciparum* strains. The biological activity studies also concluded that the phosphine ligand in complex **1** does not have any strong effect on the antiparasitic activity, unlike on the cytotoxicity, which is 10 times higher than complex **2**.

## 3. Materials and Methods

CQDP, calf thymus DNA (CT-DNA), buffers and solvents were used as received from Sigma-Aldrich Co. All other commercial reagents were also used without further purification. The NMR spectra of the compounds were recorded in CH_2_Cl_2_-*d_2_* and DMSO-*d_6_* solution in Bruker AVANCE 300 MHz or 500 MHz spectrometers. IR spectra were recorded with a Perkin Elmer Series 100 spectrometer in the 4000–400 cm^−1^ region, ultraviolet-visible (UV-Vis) spectra on a HP 8453 diode array spectrometer and fluorescence spectra on a Perkin Elmer LS45 fluorescence spectrometer. C, H and N analyses were performed using a Carlo Erba Model EA1108 elemental analyzer. ESI (+) MS spectra were obtained by direct infusion in a QTOF Bruker Impact II Mass Spectrometer in positive ion mode, utilizing CH_3_CN (LC/MS grade from Honeywell; B&J Brand) as the solvent. Electron Paramagnetic Resonance (EPR) spectra were performed on a Varian E-Line spectrometer. In the β-hematin formation inhibition assay plates were centrifuged using a Thermo Scientific IEC CL 30 centrifuge and measured in a Tecan Sunrise Absorbance Reader at 405 nm.

### 3.1. Synthesis of Copper Complexes

The synthesis of complex [Cu(CQ)_2_]Cl (**2**) was reported previously [27]. [Cu(CQ)(PPh_3_)_2_]NO_3_.2.5H_2_O (**1**): A solution of Cu(PPh_3_)_2_NO_3_ (424.7 mg; 0.65 mmol) in acetonitrile (10 mL) was stirred and refluxed until complete dissolution. An excess of CQ in 10 mL acetonitrile (250 mg, 0.78 mmol) was added to the Schlenk flask with the copper solution. The mixture was stirred and refluxed for 1 h and the initially transparent solution became yellow. The volume of the solvent was reduced under vacuum until a yellow oil was obtained. It was washed with diethyl ether and dried under vacuum resulting in a pale-yellow solid. (Yield: 475.7 mg: 72.1%). Elemental analysis (%) Calc. for C_54_H_61_ClN_4_O_5.5_P_2_Cu: C 63.9; H 6.1; N 5.5. Found: C 63.9; H 6.1; N 6.6. IR ν (N-H) 3237 cm^−1^; ν (C=C) 1613 cm^−1^; ν (C=N) 1573 cm^−1^; (NO_3_) 1394 cm^−1^. NMR-^1^H (CH_2_Cl_2_-*d_2_*; δ ppm): 8.33 (1H, d, H2), 8.09 (1H, dd, H5), 7.90 (1H, d, H8), 7.38 (24H, m, PPh_3_), 6.68 (1H, d, NH), 6.42 (1H, d, H3), 3.70 (1H, m, H1′), 2.52 (6H, m, H4′ and H5′), 1.71 (4H, m, H2′ and H3′), 1.31 (3H, d, H1″), 1.00 (3H, t, H6′); NMR-^13^C{^1^H} (CH_2_Cl_2_-*d_2_*; δ ppm): 152.43 (C2), 150.92 (C4), 150.89 (C9), 135.73 (C7), 127.70 (C8), 125.60 (C6), 123.37 (C5), 118.20 (C10), 99.75 (C3), 52.79 (C4′), 49.08 (C1′), 47.26 (C5′), 34.38 (C2′), 23.83 (C3′), 19.98 (C1″), 11.22 (C6′), PPh_3_: 133.55 (C*ortho*), 132.06 (C), 130.76 (C*para*), 129.33 (C*meta*). NMR-^31^P{^1^H} (CH_2_Cl_2_-*d_2_*; δ ppm): 0.93 (PPh_3_). λ_max_ (DMSO, nm) 262, 337. High resolution ESI (+)-MS and ESI (+)-MS-MS (acetonitrile): [M-NO_3_]^+^ (906.2937 *m*/*z*), [M-PPh_3_-NO_3_]^+^ (644.2018 *m*/*z*), [M-CQ-NO_3_]^+^ (587.1106 *m*/*z*), [CQ+H]^+^ (320.1885 *m*/*z*) and [PPh_3_+H]^+^ (160.5976 *m*/*z*). Molar conductivity in DMSO Λ_M_ = 54.92 ± 1.0 ohm^−1^ cm^2^ mol^−1^. Molecular Weight: 1015.03. Melting point: 67 °C.

### 3.2. DNA as a Target

#### 3.2.1. Spectrophotometric Titrations

UV-Vis absorption titration experiments were carried out by stepwise additions of the calf thymus DNA (CT-DNA) solution (1.38 mM, in 5 mM Tris–HCl, pH 7.54 and 50 mM NaCl buffer) to the solution of each compound (0.4 μM) in DMSO while recording the UV–Vis spectra after each addition. Native DNA absorption was subtracted by adding the same amounts of CT-DNA to the blank cell. The binding affinities (Kb_1_ and Kb_2_) were calculated from the spectrophotometric data according to the equation [47,48].

$$\frac{r}{C_{f}}=K\left(n-1\right)$$

where *r* is the number of moles of compounds bound to 1 mol of CT DNA (C_b_/C_DNA_), *n* the number of equivalent binding sites, and *K* the affinity of the complex for those sites. The concentration of free and bound compound (*C_f_* and C_b_) was calculated from *C_f_* = C (1 − α), where *C* is the total concentration of the complex, α is the mol fraction of the linked complex, calculated from α = (A_f_ − A)/(A_f_ − A_b_), where A_f_ and A_b_ are the absorbances of the free and bound complex on a specific wavelength, respectively, and *A* corresponds to absorbance of every point during the titration at the same specific wavelength. By plotting *r/C_f_* versus *r*, the interaction constant can be calculated from the slope of the curve.

#### 3.2.2. Fluorimetric Titrations

The fluorescence titrations were performed in similar manner to the UV-Vis absorption experiment. The complexes were dissolved at room temperature in a 1:5 DMSO:Buffer Tris (pH 7.4) mixture. Spectra were recorded after the addition of 10 µL aliquots of 2 mM CT-DNA. For background subtraction a blank cell with 1:5 DMSO:Buffer mixture was titrated with CT-DNA. The titration was carried out until no considerable changes were observed in the emission after the addition of the DNA aliquot. The binding affinities (Kb_1_ and Kb_2_) of the complexes were determined using the Scatchard equation described above [47].

#### 3.2.3. Viscosity Study

Viscosity measurements of solutions with different compound/DNA ratios (range 0–0.3) at a fixed CT-DNA concentration (75 µM in 5 mM Tris–HCl (pH 7.2) plus 50 mM NaCl buffer) were carried out at 25 °C in a thermostatic bath using an Ostwald viscometer. The flow time was measured with a digital stopwatch. Each measurement was repeated five times. The average value was corrected with the flow time of the solvents and represented in a graph of (η/η_0_)^1/3^ versus (metal complex]/[DNA), where η is the viscosity the CT-DNA in the presence of the complex and η_0_ is the viscosity of the CT-DNA alone [48].

#### 3.2.4. Melting Temperature Study

The melting temperature (Tm) values of the different complex/CT-DNA solutions at a molar ratio of 0.05 were measured. Absorbance of 2 mM CT-DNA solutions in Tris buffer pH 7.4 was measured at 260 nm. The temperature versus absorbance curves were obtained from the equipment software in the range of 40 to 90 °C with a heating ramp of 1 °C/min and with average intervals of one minute. The melting temperature results were expressed as differences from the Tm CT-DNA alone [49].

### 3.3. Interaction with Hemin and Inhibition of β-Hematin Formation

#### 3.3.1. Interaction with Ferriprotoporphyrin (Fe(III)PPIX) by UV-Vis Spectroscopic Titration

The association constant between the copper complexes and ferriprotoporphyrin IX (Fe(III)PPIX) was measured in triplicate using the methodology reported earlier [41]. The hemin stock solution was prepared by dissolving 3.5 mg of hemin in 10 mL DMSO. Aqueous-DMSO (40% *v*/*v*) solutions of Fe(III)PPIX (pH 7.5) (4 mM) were prepared daily by mixing 140 μL hemin stock solution with 3.86 mL DMSO, 1 mL 0.2 M Tris buffer (pH 7.5) and completed with 5 mL with double distilled deionized water. The absorbance at 402 nm was recorded. The reference cell containing 40% *v*/*v* DMSO, 0.020 M Tris pH 7.5 was also titrated with copper-chloroquine complexes to blank out the absorbance of the drug. The association constant was obtained using the equation A = (A_0_ + A_∞_K[C])/(1 + K[C]) for 1:1 complexation model using nonlinear least squares fitting [41], where A_0_ is the absorbance of hemin, A_∞_ is the absorbance of the drug–hemin adduct at saturation, A is the measured absorbance at each point of the titration, and K is the conditional association constant.

#### 3.3.2. Determination of Inhibition of β-Haematin Formation by Infrared Spectroscopy

The conversion of hemin to β-haematin was evaluated as described elsewhere [42]. A quantity of 20 mg of hemin and three equivalents of the compound of interest were dissolved in 3 mL 0.1 M NaOH solution and stirred for 30 min at 60 °C, then 0.3 mL 0.1 M HCl and 1.7 mL acetate buffer (10 M, pH 5.0) were added on that temperature. After 120 min the mixture was cooled on ice for 10 min, centrifuged and washed with water to remove acetate salts. The solid was dried, and infrared spectra recorded in KBr pallets. Primaquine and chloroquine were used as negative and positive controls, respectively [32].

#### 3.3.3. Determination of Inhibition of β-Haematin Formation by UV-Vis Spectroscopy

A 4 mM hemin chloride solution was prepared (52 mg hemin in 10 mL DMSO), that was distributed into 96 wells together with the solutions of the complexes at different concentrations (10 Mm–10μM). Acetate buffer (100 μL, 0.4 M, pH 4.4) was added to initiate hematin formation. The plates were incubated for 48 h at 37 °C, and then centrifuged for 15 min at 4000 rpm (RCF 2688). The supernatant was discarded and the solid was washed with 200 µL DMSO and centrifuged again under the same conditions as above. The solid was then dissolved in 200 µL 0.2 M NaOH. Aliquots of 100 µL were diluted with 100 µL 0.1 M NaOH for analysis by UV-Vis at 405 nm. The results were expressed as a percentage of inhibition of β-hematin [43].

### 3.4. Antiplasmodial Activity Measurements

Two strains were selected for the tests, the CQ-susceptible 3D7 strain (isolated in West Africa; obtained from MR4, Charlottesville, VA, USA), and the CQ-resistant W2 strain (isolated in Indochina; obtained from MR4, VA, USA). Both were maintained in culture in RPMI 1640 (Invitrogen, Paisley, UK), supplemented with 10% human serum (Abcys S.A., Paris, France) and buffered with 25 mM HEPES and 25 mM NaHCO_3_. Parasites were grown in A-positive human blood (Etablissement Français du Sang, Marseille, France) under controlled atmospheric conditions that consisted of 10% O_2_, 5% CO_2_ and 85% N_2_ at 37 °C with a humidity of 95%.

The two strains were synchronized twice with sorbitol before use [50], and clonality was verified every 15 days through PCR genotyping of the polymorphic genetic markers msp1 and msp2 and microsatellite loci [51,52]. Additionally, clonality was also verified each year by an independent laboratory from the Worldwide Anti-malarial Resistance Network (WWARN).

CQDP was purchased from Sigma (Saint Louis, MO, USA). CQDP was resuspended in water in concentrations ranging between 5 to 3200 nM. The synthetic compounds were resuspended in DMSO and then diluted in RPMI-DMSO (99v:1v ratio) to obtain final concentrations ranging from 0.1 nM to 100 μM.

For in vitro isotopic microtests, 25 μL/well of anti-malarial drug and 200 μL/well of the parasitized red blood cell suspension (final parasitaemia, 0.5%; final haematocrit, 1.5%) were distributed into 96 well plates. Parasite growth was assessed by adding 1 μCi of tritiated hypoxanthine with a specific activity of 14.1 Ci/mmol (Perkin-Elmer, Courtaboeuf, France) to each well at time zero. The plates were then incubated for 48 h in controlled atmospheric conditions. Immediately after incubation, the plates were frozen and thawed to lyse erythrocytes. The contents of each well were collected on standard filter microplates (Unifilter GF/B; Perkin-Elmer) and washed using a cell harvester (Filter-Mate Cell Harvester; Perkin-Elmer). Filter microplates were dried, and 25 μL of scintillation cocktail (Microscint O; Perkin-Elmer) was placed in each well. Radioactivity incorporated by the parasites was measured with a scintillation counter (Top Count; Perkin-Elmer).

IC_50_, the drug concentration able to inhibit 50% of parasite growth, was assessed by identifying the drug concentration corresponding to 50% of the uptake of tritiated hypoxanthine by the parasite in the drug-free control wells. The IC_50_ value was determined by non-linear regression analysis of log-based dose–response curves (Riasmart™, Packard, Meriden, CT, USA). IC_50_ were expressed as geometric means of 6 experiments.

### 3.5. Cell Toxicity

HepG2 or J774 cells were planted (1.0 × 10^4^ per well) in 100 mL of RPMI or DMEM, respectively, in 96-well plates. The drugs were introduced in a volume of 100 mL suspended in the medium 24 h later, and the plates were incubated for 72 h at 37 °C and 5% CO_2_. Drugs were tested in triplicate at eight different doses (160–2.5 µM). The positive control was gentian violet (Synth, Diadema, São Paulo, Brazil), the negative control was the use of untreated cells. The plates were then incubated for 4–6 h with 20 mL of AlamarBlue (Life). A SpectraMA × 190 instrument (Molecular Devices, Sunnyvale, CA, USA) was used to take colorimetric values at 570 and 600 nm. Three independent experiments were used to obtain the mean CC_50_ values.

## 4. Conclusions

A new copper(I)-CQ complex, [Cu(CQ)(PPh_3_)_2_]NO_3_ (**1**), was synthesized and characterized. The effect of compound **1** and [Cu(CQ)_2_]Cl (**2**) on the identified essential targets for the parasite survival i.e., inhibition of DNA synthesis and hemozoin formation, was studied. Both Cu(I)-CQ complexes displayed similar intercalator properties with the DNA to CQ alone. Their interaction with hemin and the inhibition of β-hematin formation were also similar to CQDP. As well, this interaction was potentialized by the incorporation of the metal center. In in vitro assays, complex **1** demonstrated higher activity against both the CQ-susceptible (3D7) and CQ-resistant (W2) *P. falciparum* strains, which makes it a potential antimalarial drug.

## Figures and Tables

**Table 1:** Binding constants for the interaction between copper complexes and calf thymus DNA, and DNA thermal denaturation data.

Compounds	Absorption Titration		Emission Titration		DNA Thermal Denaturation	
	Kb_1_ (×10^7^ M^−1^) ^a^	Kb_2_ (×10^5^ M^−1^) ^a^	Kb_1_ (×10^7^ M^−1^) ^b^	Kb_2_ (×10^5^ M^−1^) ^b^	T_m_ °C *	ΔT_m_ °C **
[Cu(CQ)(PPh_3_)_2_]NO_3_ (**1**)	1.20 ± 0.35	3.15 ± 0.51	4.66 ± 0.03	13.8 ± 0.47	80.4 ± 0.1	−0.3
[Cu(CQ)_2_]Cl (**2**)	2.28 ± 0.01	4.58 ± 0.50	1.58 ± 0.56	12.8 ± 4.16	80.4 ± 0.1	−0.3
CQDP	1.07 ± 0.05	1.94 ± 0.19	5.61 ± 1.46	3.14 ± 1.53	80.7 ± 0.1	0.0

Chloroquine diphosphate (CQDP), Chloroquine (CQ). ^a^ Average values calculated at 343 nm from absorption spectra. ^b^ Average values calculated at 384 nm from fluorescence spectra.* Thermal denaturation curves of CT DNA in presence of the compounds individually at pH 7.4 (Tris–HCl buffer 5 mM NaCl) at Ri = 0.05. ** Temperature difference from the free CT DNA (Tm = 80.7 °C) under the same condition.

**Table 2:** Association constants (LogK) of copper-CQ complexes and CQDP with hemin, and characteristics of inhibition of β-hematin formation.

Compound	Log K	Inhibition of β-Hematin Formation ^a^	HAI_50_ (mM) in Buffer ^b^
**1**	5.33 ± 0.10	+	0.51 ± 0.01 (1.3)
**2**	3.55 ± 0.06	+	0.66 ± 0.03 (1.0)
CQDP	5.35 ± 0.03	+	0.64 ± 0.01 (1.0)

HAI_50_ is the compound-to-hemin ratio required to inhibit 50% of heme aggregation against a control experiment. Values in parentheses are the relative activity with respect to CQDP. ^a^ After 2 h reaction. ^b^ After 48 h reaction.

**Table 3:** Biological activity of copper-CQ complexes and CQDP against *Plasmodium falciparum* (3D7 and W2 strains), mammalian cell cytotoxicity and activity indexes.

	CQ-Susceptible Strain (3D7)		CQ-Resistant Strain (W2)		Susceptible Index	Resistance Index	Cell J744	Selectivity Index (SI)	
Compound	IC_50_ (nM)	Relative activity	IC_50_ (nM)	Relative activity	3D7 IC_50_/ W2 IC_50_	W2 IC_50_/ 3D7 IC_50_	CC_50_ (µM)	J744 CC_50_/ 3D7 IC_50_	J744 CC_50_/ W2 IC_50_
Chloroquine	8	--	404	--	0.02	50.5	35.1 ± 11.0	4387	86.9
[Cu(CQ)(PPh_3_)_2_]NO_3_	6	1.33	231	1.75	0.03	38.5	2.9 ± 0.8	483	12.6
Cu(CQ)_2_Cl	13	0.62	279	1.45	0.05	21.5	29.7 ± 7.0	2284	106.4
Gentian Violet	--	--	--	--	--	--	1.0 ± 0.03	--	--

Relative activity = Chloroquine IC_50_/Copper complex IC_50_. Resistance Index (W2 IC_50_ (CQ-resistant strains)/3D7 IC_50_ (CQ-susceptible strains)) and Sensitive Index (3D7 IC_50_ (CQ-susceptible strains)/W2 IC_50_ (CQ-resistant strains)).

## Data Availability

The data presented in this study are available on request from the corresponding author. Data are contained within the article.

## Supplementary Materials

The following supporting information can be downloaded at: https://www.mdpi.com/article/10.3390/ph15080921/s1. Figures S1–S5: ^1^H, ^13^C{^1^H},^1^H-^1^H COSY, ^1^H-^13^C HMQC and ^1^H-^13^C HMBC NMR spectrum of [Cu(CQ)(PPh_3_)_2_]NO_3_ (**1**) in CH_2_Cl_2_-*d_2_* at 298 K. Figure S6: ^31^P{^1^H} NMR spectrum of [Cu(CQ)(PPh_3_)_2_]NO_3_ (**1**) in CH_2_Cl_2_-*d_2_* at 298 K. Table S1: Chemical shifts of the protons and carbons for the complex **1** and their variations when compared with the free chloroquine ligand. Figure S7: Theoretical (a) and Experimental (b) isotopic distribution for [Cu(CQ)(PPh_3_)_2_]^+^. Figure S8: EPR spectra for (a) [Cu(PPh_3_)_2_(NO_3_)], (b) [Cu(CQ)(PPh_3_)_2_]NO_3_ and (c) Cu(II) complex for comparison. Figure S9: ^1^H NMR spectra of the complex **2** in 90% DMSO-*d_6_* and 10% PBS-*D*_2_*O*, obtained at 0–48 h after sample preparation. [Complex **2**] = 13.9 mM. Figure S10: ^1^H NMR spectra of the complex **1** in 90% DMSO-*d_6_* and 10% PBS-*D_2_O*, obtained at 0–48 h after sample preparation. [Complex **1**] = 9.2 mM. Figure S11: ^31^P{^1^H} NMR spectra of the complex **1** in 90% DMSO-*d_6_* and 10% PBS-*D_2_O*, obtained at 0–48 h after sample preparation. [Complex **1**] = 9.2 mM. Figure S12: Absorption spectra of the complexes **1**(a) and **2**(b) in 10% DMSO and 90% PBS, obtained at 0–48 h after sample preparation. [Complex] = 10 μM.
