# Supplementary material for: Copper (I)-Chloroquine Complexes: Interactions with DNA and Ferriprotoporphyrin, Inhibition of β-Hematin Formation and Relation to Antimalarial Activity
Source: Pharmaceuticals (Basel). 2022 Jul 25;15(8):921. doi: 10.3390/ph15080921 (PMC9329717; doi:10.3390/ph15080921)
Supplement: Supplementary file 1 [file pharmaceuticals-15-00921-s001.zip › pharmaceuticals-1779742-supplementary.pdf]

Copper(I)-chloroquine complexes: Interaction with DNA and Ferriprotoporphyrin, Inhibition of β-hematin Formation and Relation to Antimalarial Activity

Wilmer Villarreal [1,2], William Castro [2], Sorenlis González [2], Marylin Madamet [3,4,5,6], Rémy Amalvict [3,4,5,6], Bruno Pradines [3,4,5,6], Maribel Navarro [2,7]*

[1] Grupo de Química Inorgânica Medicinal e Reações Aplicadas. Instituto de Química, Universidade Federal do Rio Grande do Sul (UFRGS), Porto Alegre 91501-970, Brazil.

[2] Centro de Química, Instituto Venezolano de Investigaciones Científicas (IVIC). Caracas 1020-A, Venezuela.

[3] Unité Parasitologie et Entomologie, Département Microbiologie et Maladies Infectieuses, Institut de Recherche Biomédicale des Armées, Marseille 13005, France.

[4] Aix Marseille Unive, IRD, SSA, AP-HM, VITROME, Marseille 13005, France.

[5] IHU Méditerranée Infection, Marseille 13005, France.

[6] Centre National de Référence du Paludisme, Marseille 13005, France.

[7] Laboratório de Química Bioinorgânica e Catálise. Departamento de Química, Instituto de Ciências Exatas, Universidade Federal de Juiz de Fora (UFJF), Juiz de Fora 36036-900, Brazil.

Supporting Information

Figure S1 – S5: ^{1}H, $^{13}C\{^{1}H\}$, ^{1}H-^{1}H COSY, ^{1}H-^{13}C HMQC and ^{1}H-^{13}C HMBC NMR spectrum of $[Cu(CQ)(PPh_3)_2]NO_3$ (**1**) in CD_2Cl_2 at 298 K.
Figure S6: $^{31}P\{^{1}H\}$ NMR spectrum of $[Cu(CQ)(PPh_3)_2]NO_3$ (**1**) in CD_2Cl_2 at 298 K.
Table S1: Chemical shifts of the protons and carbons for the complex **1** and their variations when compared with the free chloroquine ligand.
Figure S7: Theoretical (a) and Experimental (b) isotopic distribution for $[Cu(CQ)(PPh_3)_2]^+$.
Figure S8: EPR spectra for (a) $[Cu(PPh_3)_2(NO_3)]$, (b) $[Cu(CQ)(PPh_3)_2]NO_3$ and (c) Cu(II) complex for comparison.
Figure S9: ^{1}H NMR spectra of the complex **2** in 90% DMSO-*d6* and 10% PBS-*D2O*, obtained at 0 – 48 h after sample preparation. [Complex **2**] = 13.9 mM.
Figure S10: ^{1}H NMR spectra of the complex **1** in 90% DMSO-*d6* and 10% PBS-*D2O*, obtained at 0 – 48 h after sample preparation. [Complex **1**] = 9.2 mM.
Figure S11: $^{31}P\{^{1}H\}$ NMR spectra of the complex **1** in 90% DMSO-*d6* and 10% PBS-*D2O*, obtained at 0 – 48 h after sample preparation. [Complex **1**] = 9.2 mM.
Figure S12: Absorption spectra of the complexes **1**(a) and **2**(b) in 10% DMSO and 90% PBS, obtained at 0 – 48 h after sample preparation. [Complex] = 10 μM.

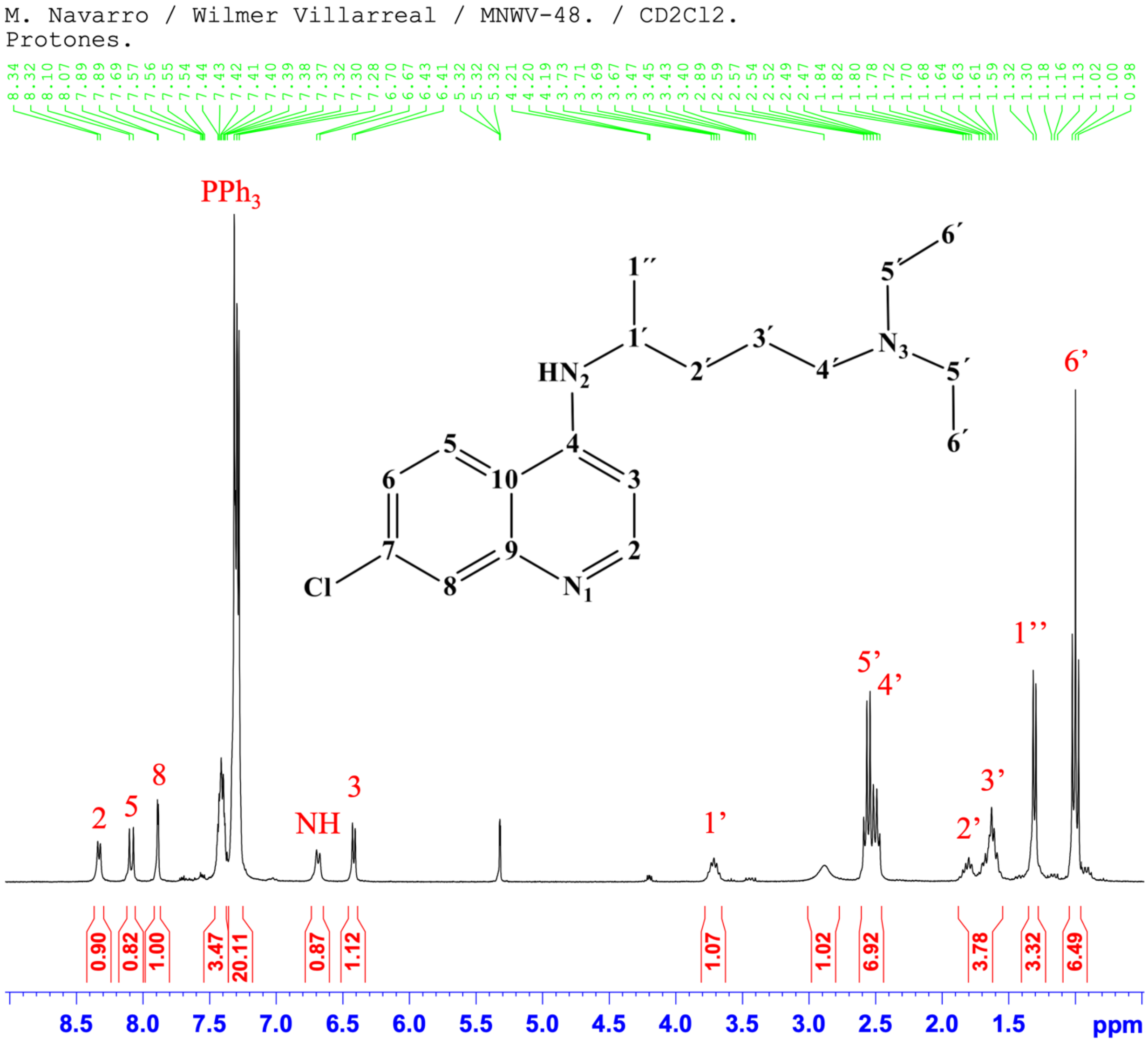

Figure S1 – The ^{1}H NMR spectrum of $[Cu(CQ)(PPh_3)_2]NO_3$ (**1**) in CD_2Cl_2 at 298 K

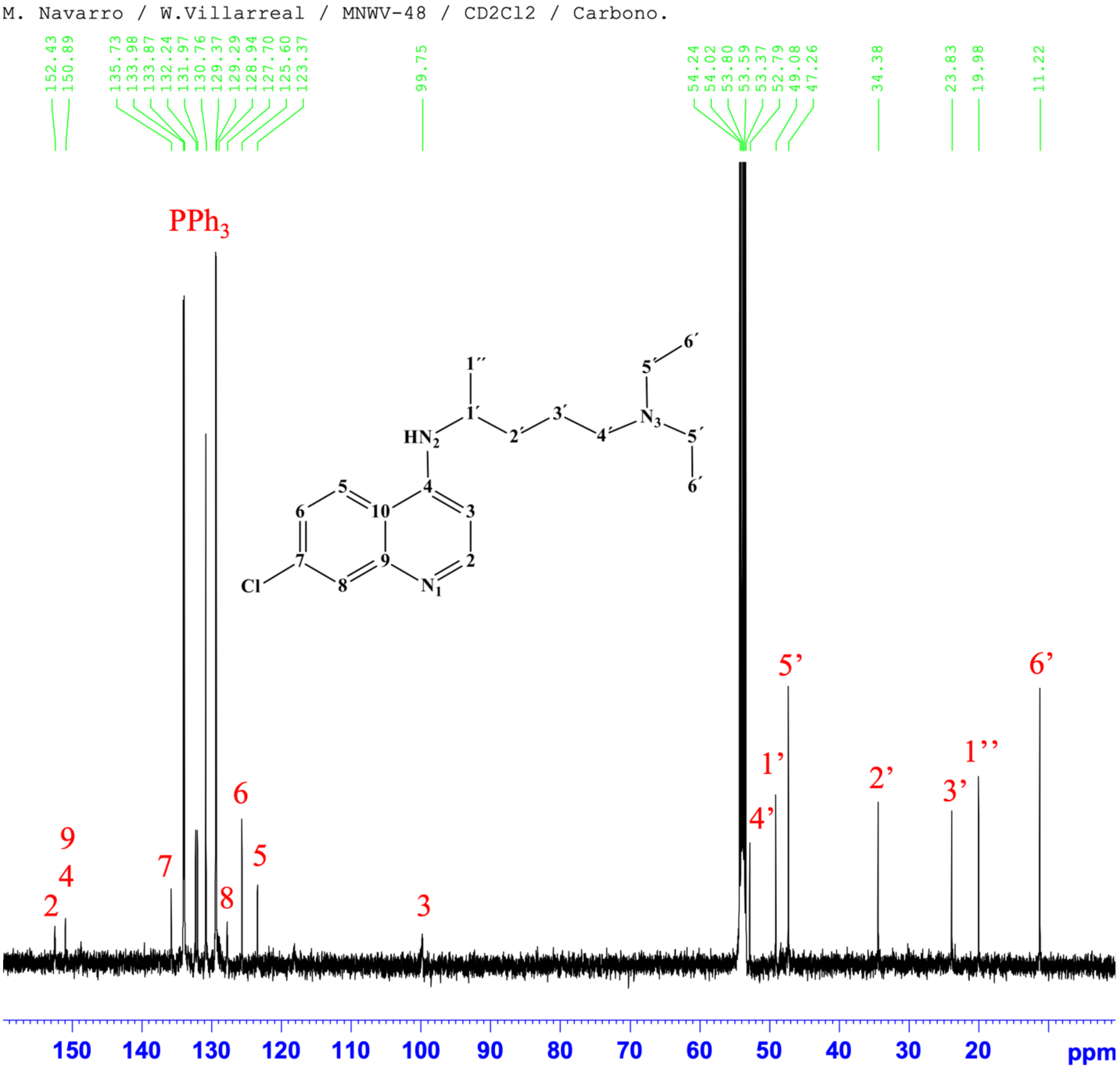

Figure S2 – The $^{13}C\{^{1}H\}$ NMR spectrum of $[Cu(CQ)(PPh_3)_2]NO_3$ (**1**) in CD_2Cl_2 at 298 K

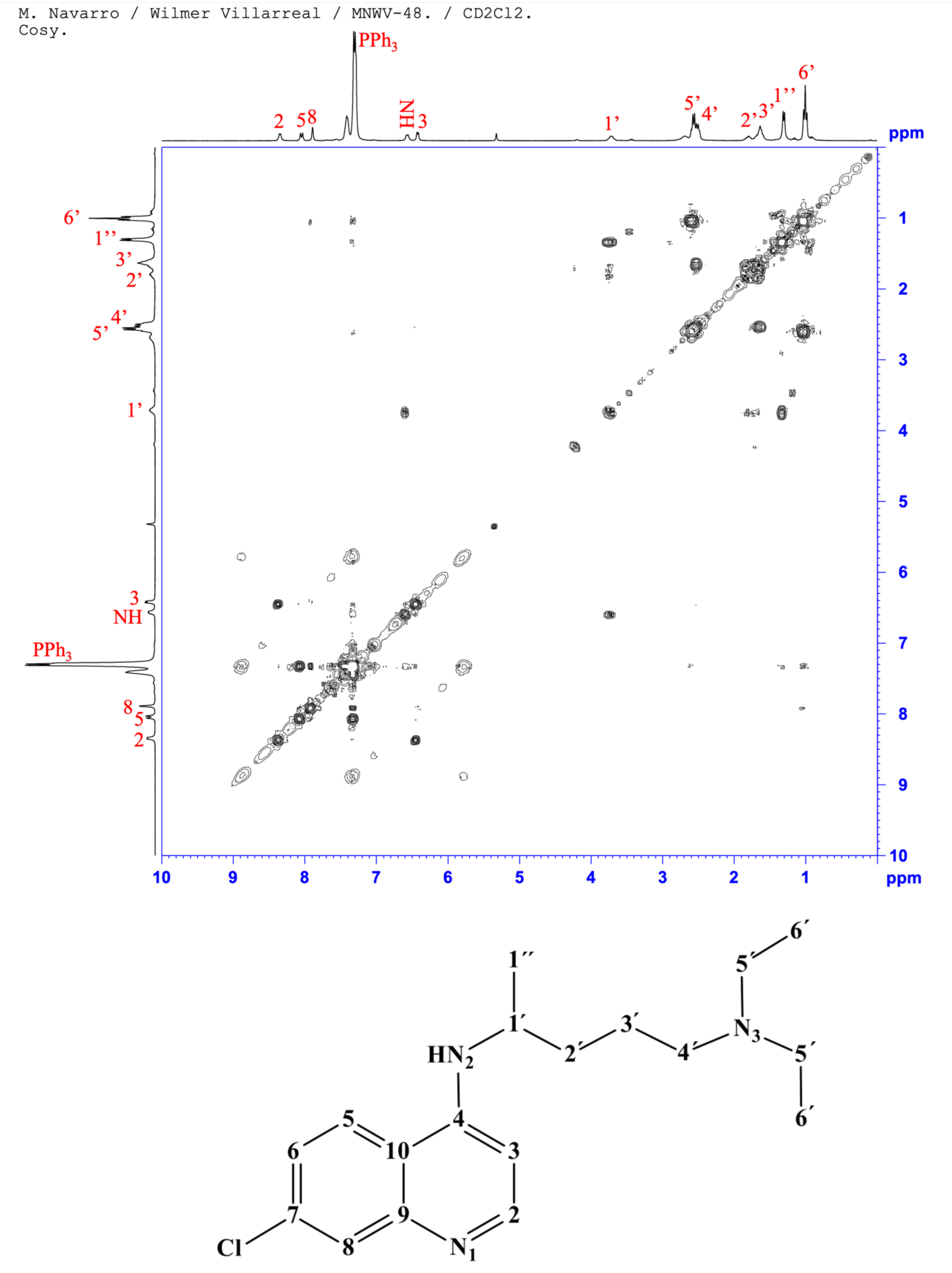

Figure S3 – The 2D homonuclear ^{1}H-^{1}H COSY NMR spectrum of $[Cu(CQ)(PPh_3)_2]NO_3$ (**1**) in CD_2Cl_2 at 298 K

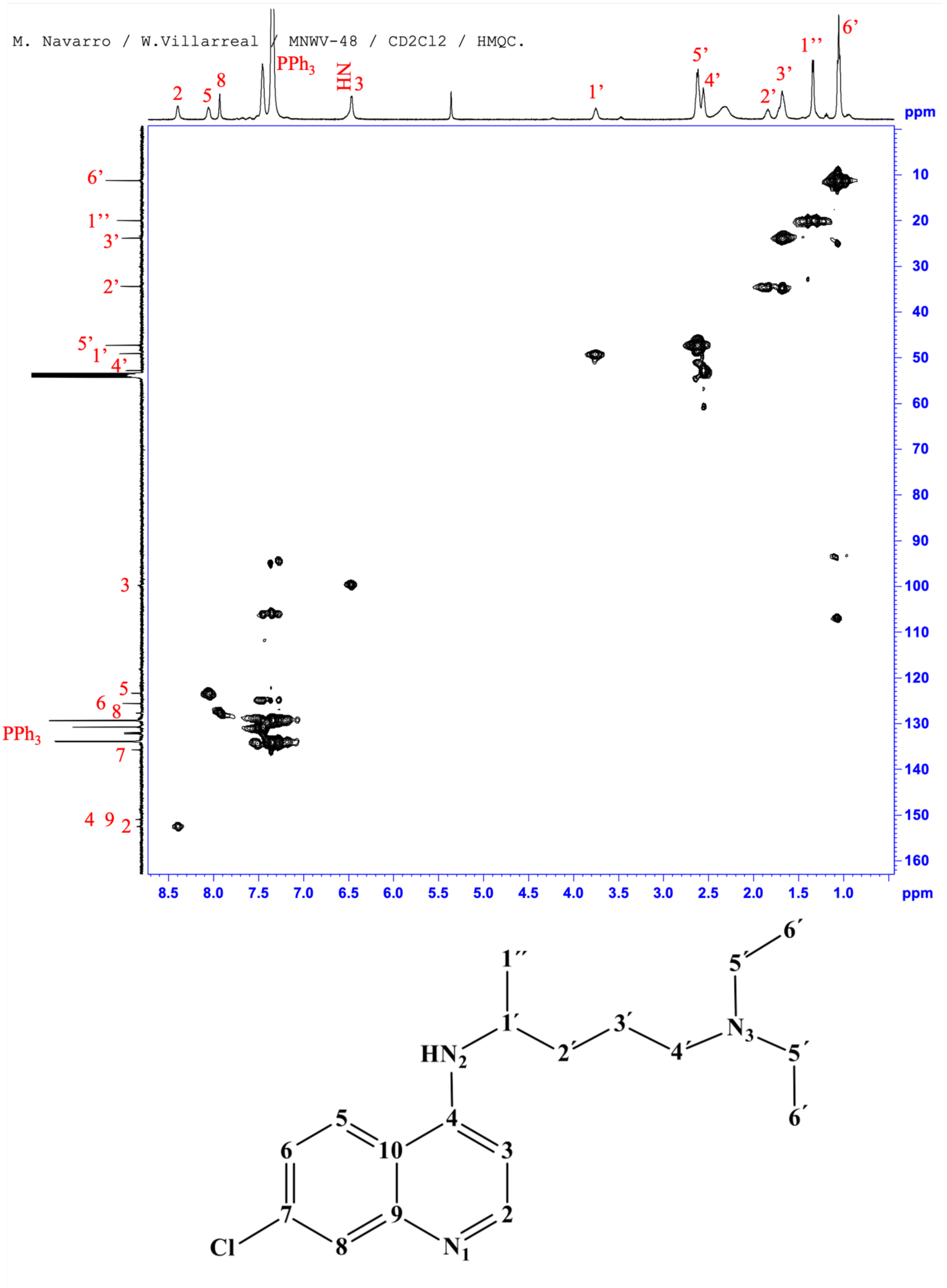

Figure S4 – The 2D heteronuclear ^{1}H-^{13}C HMQC NMR spectrum of $[Cu(CQ)(PPh_3)_2]NO_3$ (**1**) in CD_2Cl_2 at 298 K

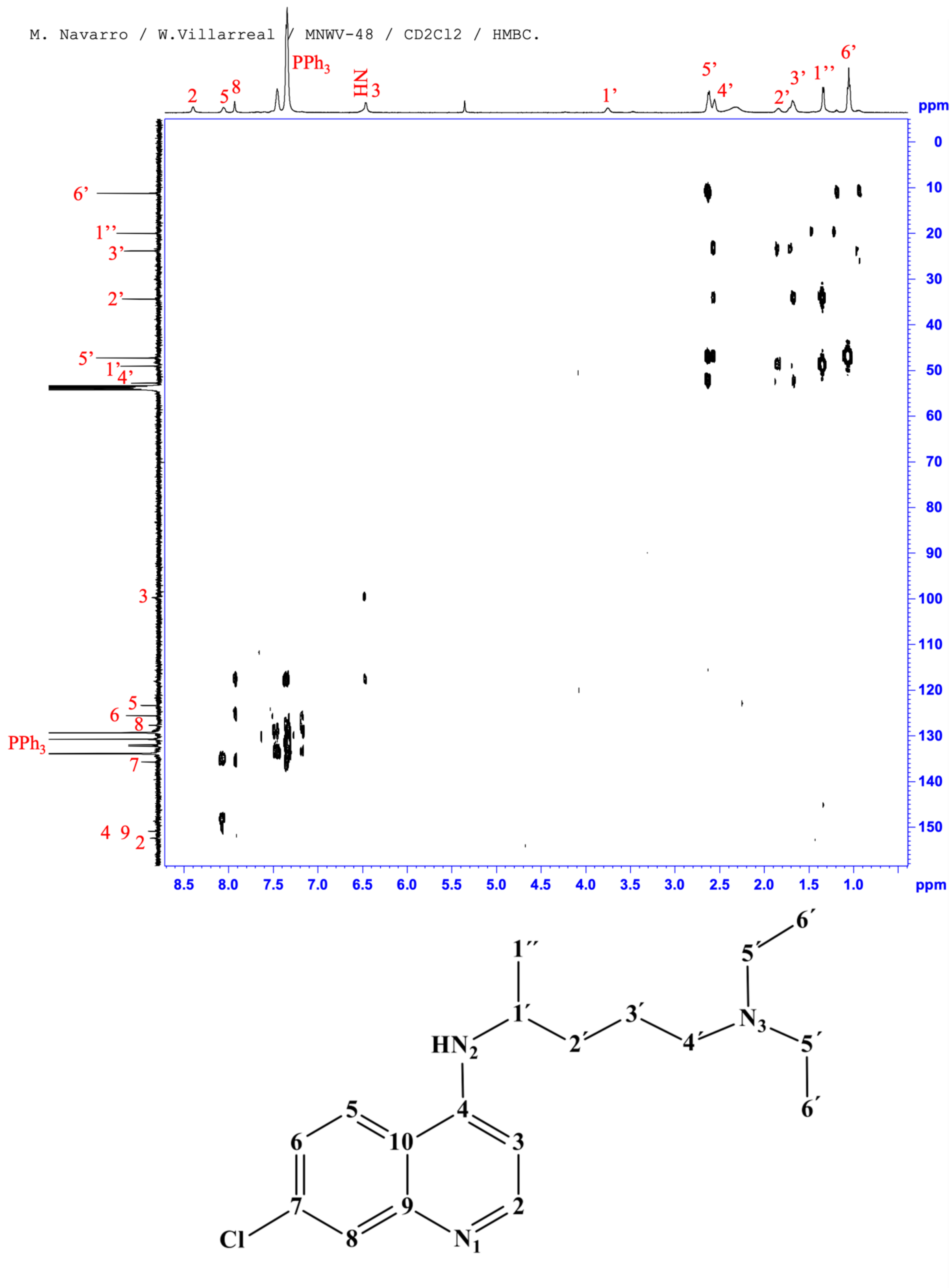

Figure S5 – The 2D heteronuclear ^1H-^{13}C HMBC NMR spectrum of $[Cu(CQ)(PPh_3)_2]NO_3$ (**1**) in CD_2Cl_2 at 298 K

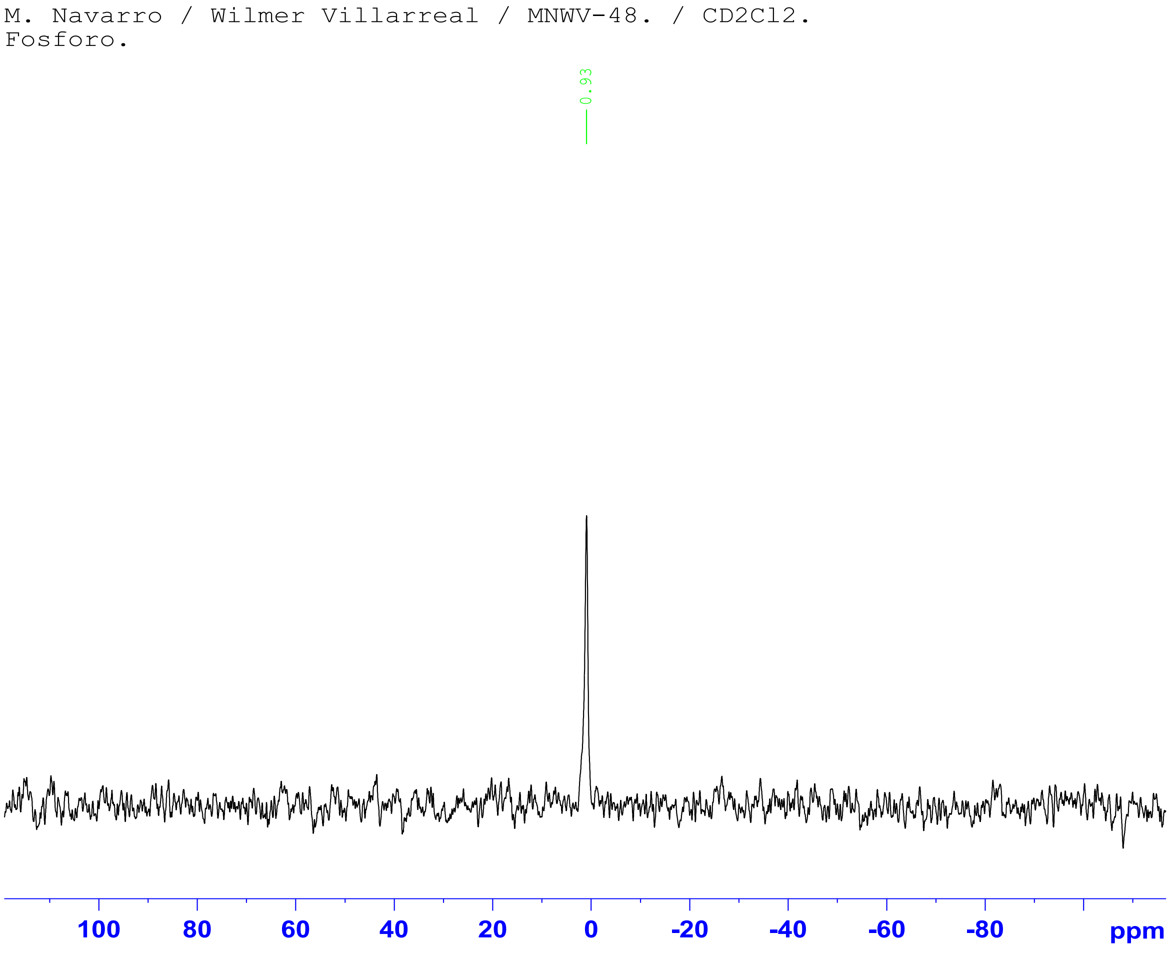

Figure S6 - $^{31}P\{^{1}H\}$ NMR spectrum of $[Cu(CQ)(PPh_3)_2]NO_3$ (**1**) in CD_2Cl_2 at 298 K

Table S1 - Chemical shifts of the protons and carbons for the complex **1** and their variations when compared with the free chloroquine ligand

Chemical shifts in ^{1}H NMR (CH_2Cl_2-d_2)			Chemical shifts in $^{13}C\{^{1}H\}$ NMR (CH_2Cl_2-d_2)		
Position	δ (ppm)	Δ (ppm)	Position	δ (ppm)	Δ (ppm)
6'	1.00	0.02	6'	11.22	0.40
1"	1.31	0.01	1"	19.98	0.22
2' and 3'	1.71	0.06	3'	23.83	0.36
4' and 5'	2.47	0.07	2'	34.38	0.39
1'	3.70	0.01	5'	47.26	0.08
NH	6.68	1.27	1'	49.08	0.33
3	6.42	0.04	4'	52.79	0.07
PPh_3	7.38	------	3	99.75	0.08
5	8.09	0.72	10	118.20	0.32
8	7.90	0.01	5	123.37	1.23
2	8.33	0.13	6	125.60	0.6
			8	127.70	1.11
			b	129.33	----
			a	130.76	----
			d	132.06	----
			c	133.55	----
			7	135.73	0.93
			9	150.89	1.24
			4	150.92	1.11
			2	152.43	0.15

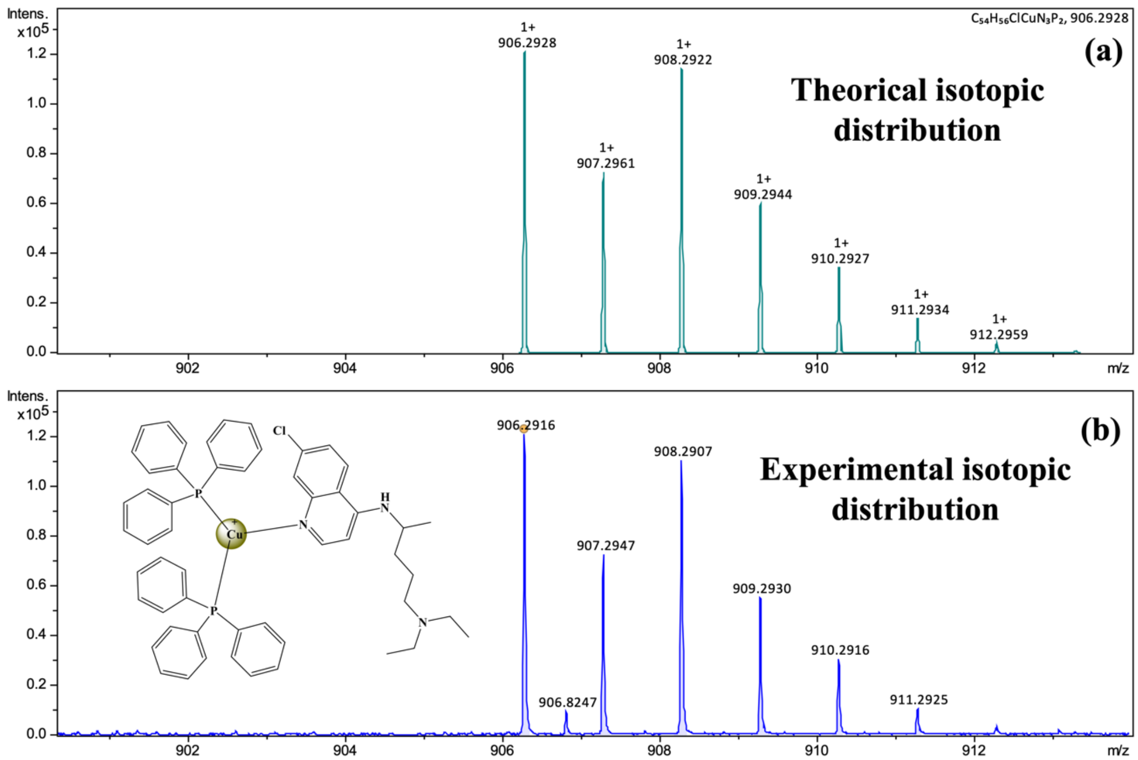

Figure S7 - Theoretical (a) and Experimental (b) isotopic distribution for $[Cu(CQ)(PPh_3)_2]^+$.

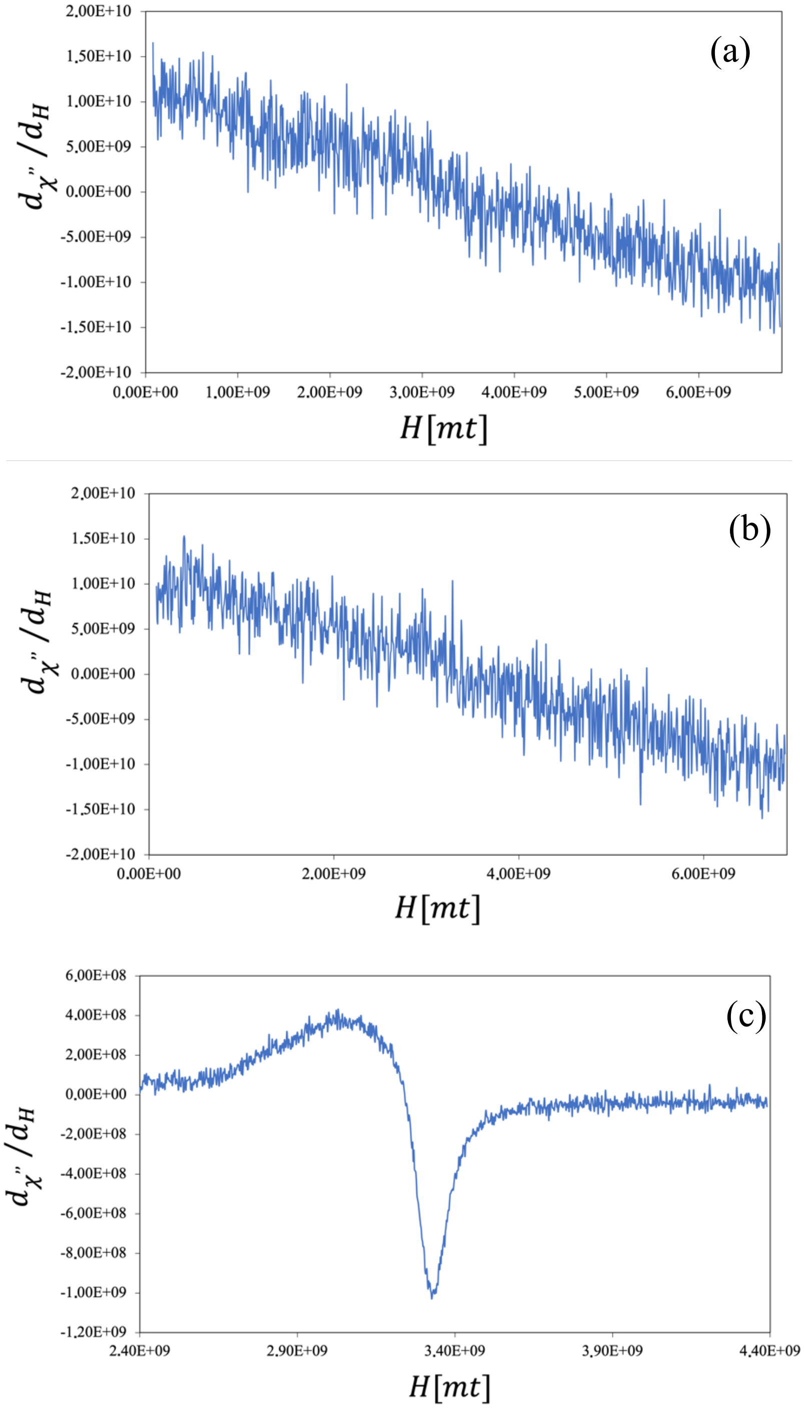

Figure S8 – EPR spectra for (a) [Cu(PPh_3)$_2$(NO_3)], (b) [Cu(CQ)(PPh_3)$_2$]NO_3 and (c) Cu(II) complex for comparison.

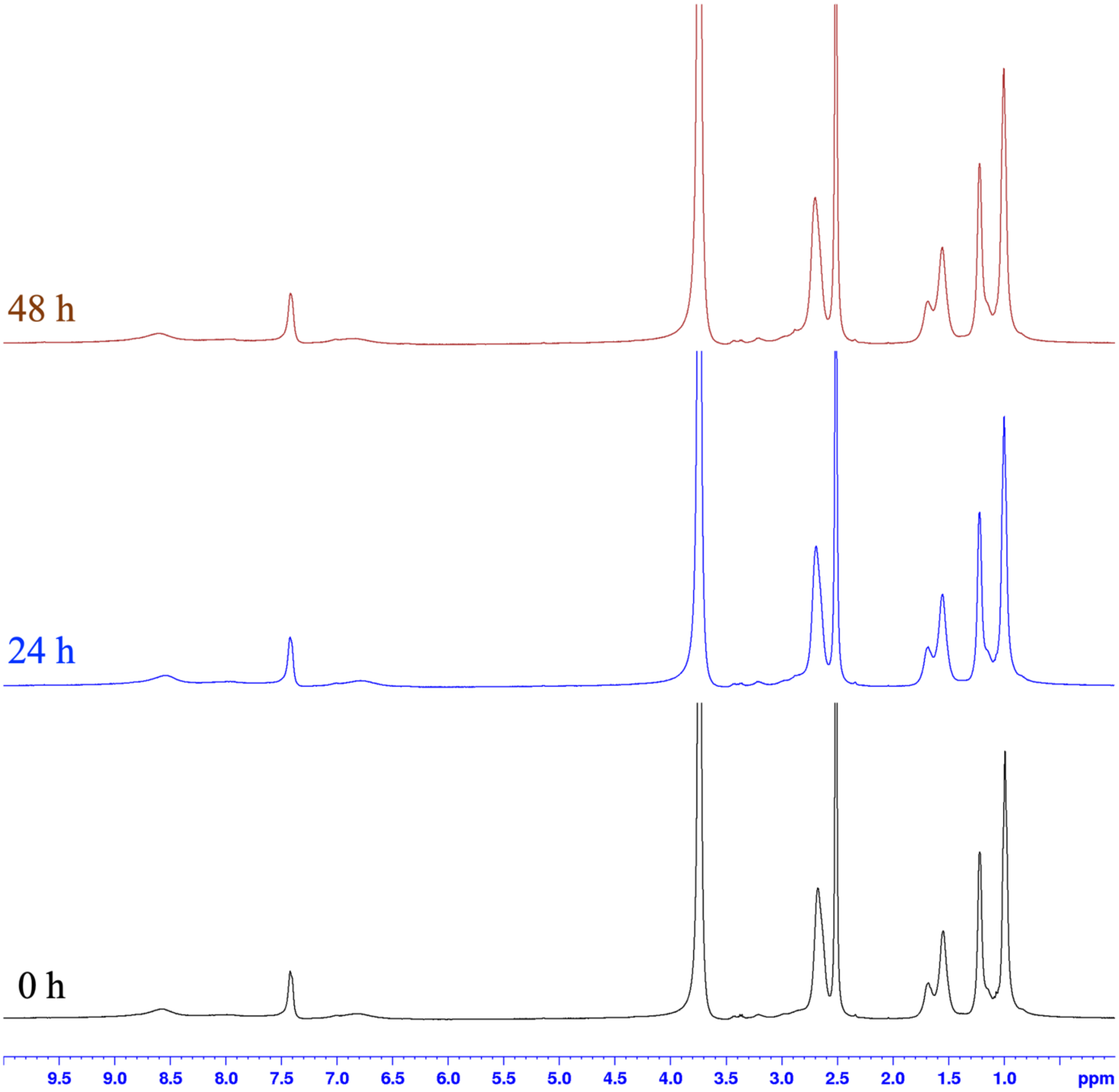

Figure S9 – ^{1}H NMR spectra of the complex **2** in 90% DMSO-d_6 and 10% PBS-D_2O, obtained at 0 – 48 h after sample preparation. [Complex **2**] = 13.9 mM.

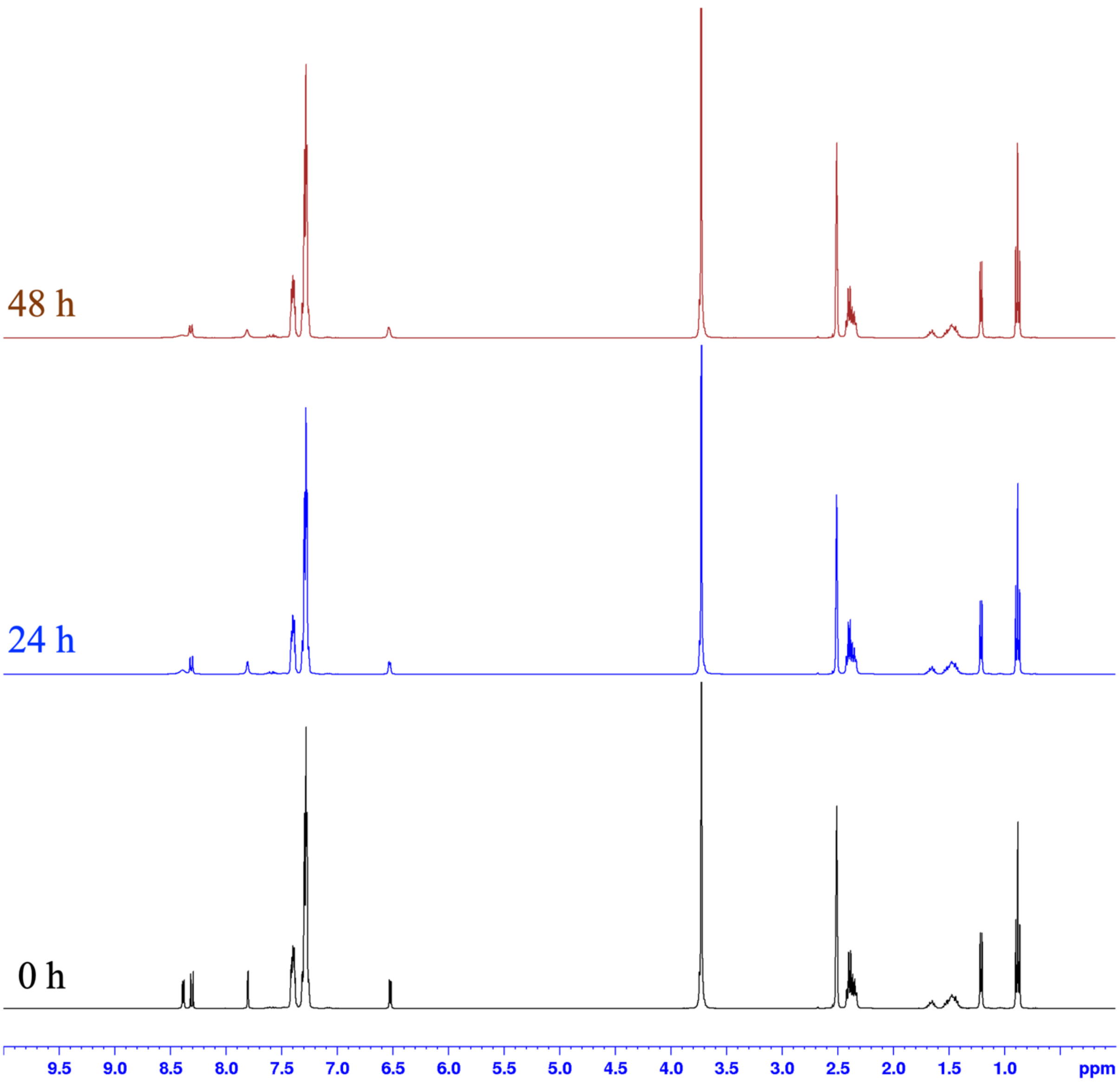

Figure S10 – ^{1}H NMR spectra of the complex **1** in 90% DMSO-d_6 and 10% PBS-D_2O, obtained at 0 – 48 h after sample preparation. [Complex **1**] = 9.2 mM.

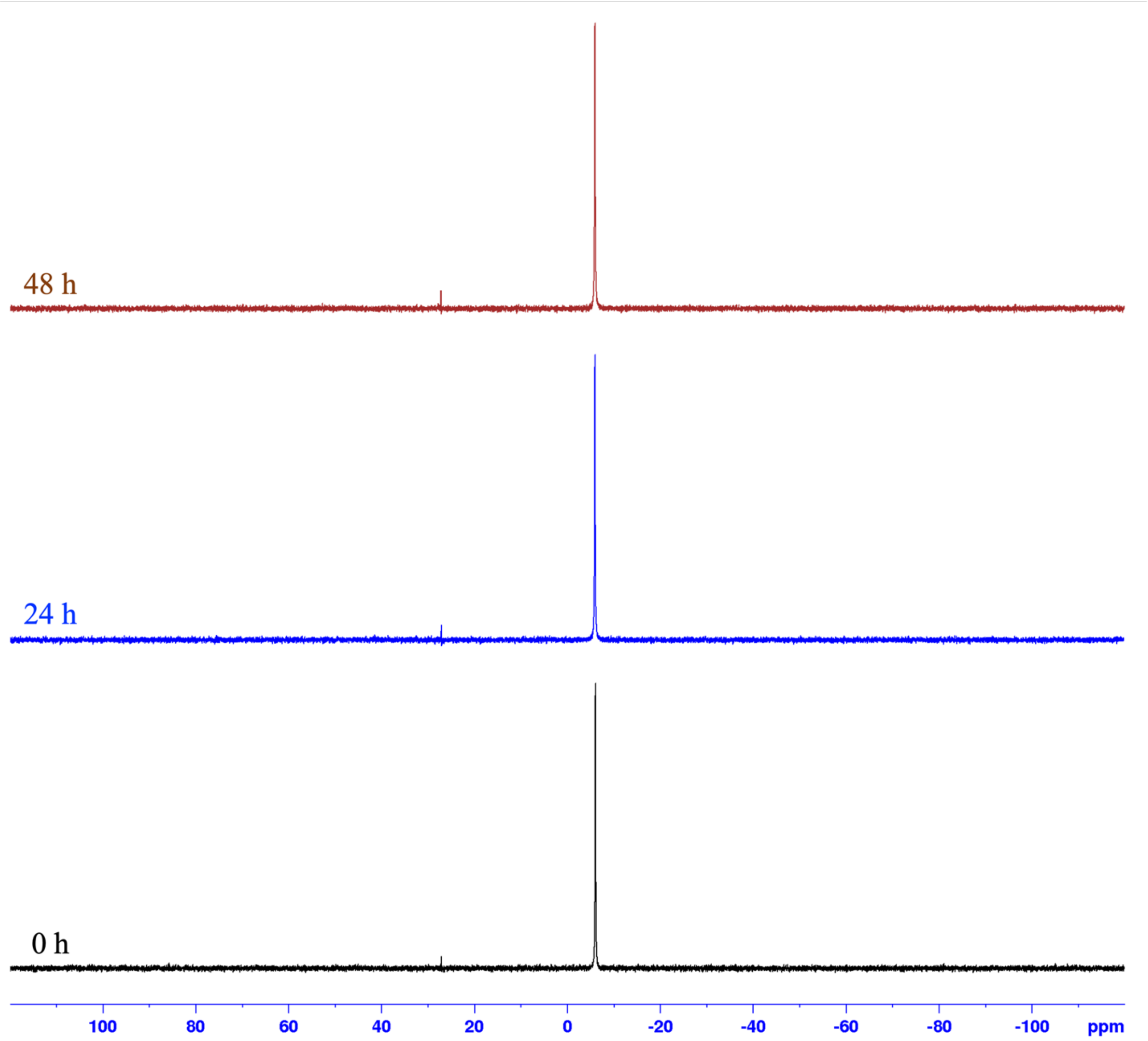

Figure S11 – $^{31}P\{^1H\}$ NMR spectra of the complex **1** in 90% DMSO-d_6 and 10% PBS-D_2O, obtained at 0 – 48 h after sample preparation . [Complex **1**] = 9.2 mM.

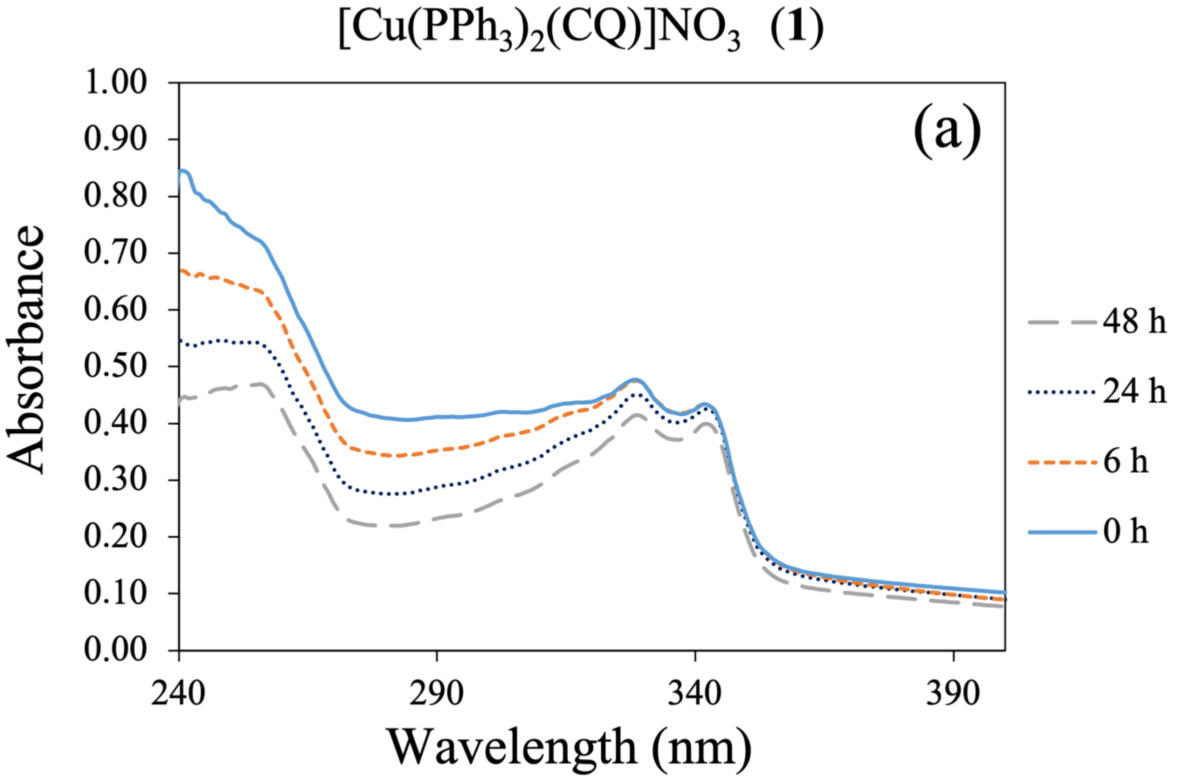

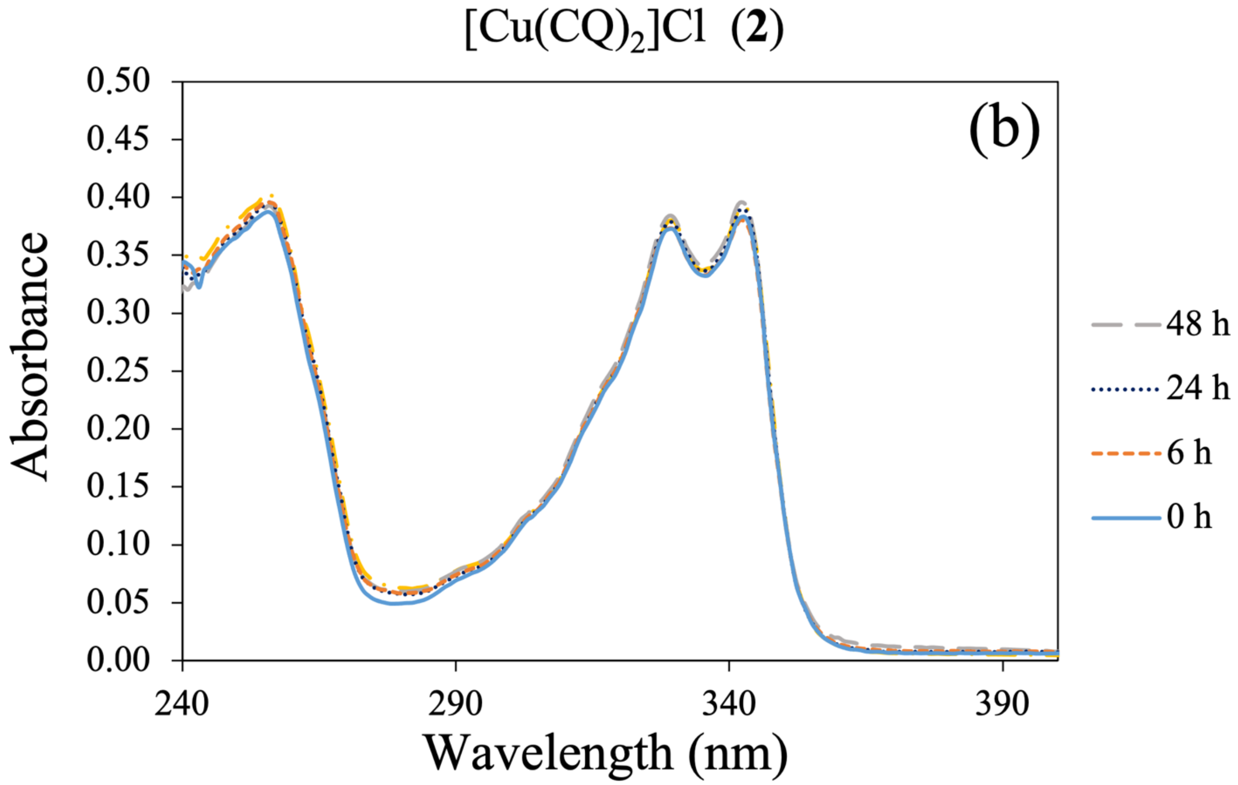

Figure S12 – Absorption spectra of the complexes **1**(a) and **2**(b) in 10% DMSO and 90% PBS, obtained at 0 – 48 h after sample preparation . [Complex] = 1 μM.
